# CARD14 signalosome formation is associated with its endosomal relocation and mTORC1-induced keratinocyte proliferation

**DOI:** 10.1042/BCJ20240058

**Published:** 2024-09-06

**Authors:** Paul A. O'Sullivan, Aigerim Aidarova, Inna S. Afonina, Joan Manils, Teresa L. M. Thurston, Rachael Instrell, Michael Howell, Stefan Boeing, Sashini Ranawana, Melanie B. Herpels, Riwia Chetian, Matilda Bassa, Helen Flynn, David Frith, Ambrosius P. Snijders, Ashleigh Howes, Rudi Beyaert, Anne M. Bowcock, Steven C. Ley

**Affiliations:** 1 The Francis Crick Institute, London NW1 1AT, U.K.; 2 Institute of Immunity and Transplantation, University College London, London NW3 2PP, U.K.; 3 VIB Center for Inflammation Research and Department of Biomedical Molecular Biology, Ghent University, Ghent, Belgium; 4 Immunology Unit, Department of Pathology and Experimental Therapy, School of Medicine, University of Barcelona, Barcelona, Spain; 5 MRC Centre for Molecular Bacteriology and Infection, Imperial College London, London SW7 2AZ, U.K.; 6 National Heart and Lung Institute, Imperial College London, London W12 0NN, U.K.; 7 Department of Oncological Science, Dermatology, and Genetics and Genome Sciences, Icahn School of Medicine at Mount Sinai, New York 10029, U.S.A.

**Keywords:** a20, ABIN1, CARD14, keratinocytes, LUBAC, mTOR, NF-κB, psoriasis

## Abstract

Rare mutations in *CARD14* promote psoriasis by inducing CARD14-BCL10-MALT1 complexes that activate NF-κB and MAP kinases. Here, the downstream signalling mechanism of the highly penetrant CARD14^E138A^ alteration is described. In addition to BCL10 and MALT1, CARD14^E138A^ associated with several proteins important in innate immune signalling. Interactions with M1-specific ubiquitin E3 ligase HOIP, and K63-specific ubiquitin E3 ligase TRAF6 promoted BCL10 ubiquitination and were essential for NF-κB and MAP kinase activation. In contrast, the ubiquitin binding proteins A20 and ABIN1, both genetically associated with psoriasis development, negatively regulated signalling by inducing CARD14^E138A^ turnover. CARD14^E138A^ localized to early endosomes and was associated with the AP2 adaptor complex. AP2 function was required for CARD14^E138A^ activation of mTOR complex 1 (mTORC1), which stimulated keratinocyte metabolism, but not for NF-κB nor MAP kinase activation. Furthermore, rapamycin ameliorated CARD14^E138A^-induced keratinocyte proliferation and epidermal acanthosis in mice, suggesting that blocking mTORC1 may be therapeutically beneficial in CARD14-dependent psoriasis.

## Introduction

Psoriasis is a chronic inflammatory skin disease affecting 2–3% of the ancestral European population and is characterized by keratinocyte proliferation, altered keratinocyte differentiation, defective skin barrier formation, angiogenesis and immune cell infiltration in the dermis and epidermis [1]. A serious systemic inflammatory disorder, psoriasis is associated with multiple co-morbidities, including psoriatic arthritis, inflammatory bowel disease, metabolic syndrome and cardiovascular disease [2].

Both genetic and environmental risk factors are involved in the development of psoriasis. Genome-wide association studies (GWAS) in humans have revealed over 70 genetic loci that individually confer low risk for disease [3,4]. Candidate genes within these loci highlight the importance of both innate immune system function and keratinocyte differentiation in psoriasis pathogenesis. We have previously shown that rare, gain-of-function and highly penetrant (>90%) alterations within the *Caspase recruitment domain-containing protein 14 (CARD14)* gene can promote the development of psoriasis vulgaris (plaque psoriasis) and psoriatic arthritis [5,6]. In addition, a common variant of *CARD14* (c.C2458T/p.R820W) exceeds genome-wide significance for association with psoriasis, indicating a general role for CARD14 in psoriasis pathogenesis [5,7]. Mutations in *CARD14* have also been identified as causative factors in familial pityriasis rubra pilaris, a rare papulosquamous disorder phenotypically related to psoriasis [8].

CARD14 is a member of the CARMA (CARD-CC) family of scaffold proteins, which includes CARD10 and CARD11 [9]. Each of these proteins has a similar structure, comprising an N-terminal CARD domain, followed by a LATCH linker region, a coiled-coil (CC) domain, and a C-terminal MAGUK domain (PDZ-SH3-GUK). Most knowledge on downstream signalling has been obtained in relation to CARD11, which shares ∼30% overall amino acid identity with CARD14 [10]. CARD11 is required for T cell antigen-receptor (TCR) activation of NF-κB transcription factors [11]. In resting T cells, CARD11 signalling activity is prevented by an inhibitory domain (ID) that interacts with the CARD and CC domains, blocking interaction with downstream signalling proteins [12]. Following TCR stimulation, the CARD11 ID is phosphorylated by protein kinase C (PKC) θ, inducing a conformational change that allows CARD11 to bind to BCL10 (B cell lymphoma protein 10) and the paracaspase MALT1 (Mucosa-associated lymphoid tissue lymphoma translocation protein 1). Ubiquitination of the BCL10 and MALT1 components of the CARD11-BCL10-MALT1 (CBM) complex promotes the recruitment of the IκB kinase (IKK) complex, via its ubiquitin-binding NEMO subunit [13]. Simultaneous ubiquitin-mediated recruitment of the TAK1-TAB2 complex facilitates TAK1-mediated phosphorylation of the activation loops of CBM-associated IKK, leading to activation of NF-κB [12]. TAK1 also phosphorylates and activates MAP 2-kinases promoting activation of MAP kinases.

CARD11 signalling is also activated by mutation in diffuse large B cell lymphoma. Oncogenic *CARD11* mutations are found in the CARD, LATCH and CC regions [14,15], disrupting ID-mediated auto-inhibition and bypassing the requirement for receptor-induced phosphorylation to activate CARD11 signalling. Consequently, oncogenic CARD11 variants constitutively form CBM complexes that stimulate NF-κB activity to promote lymphoma proliferation and survival. Psoriasis-associated mutations in CARD14 similarly cluster within the CARD and CC domains [16] and we have recently demonstrated that the highly penetrant *CARD14*^E138A^ psoriasis-associated mutation induces a conformational change in CARD14 that induces interaction with BCL10 and MALT1 [17,18]. This complex triggers activation of IKK/NF-κB and MAP kinases in keratinocytes and stimulates the transcription of several key psoriasis-associated cytokine and chemokine genes in the absence of exogenous stimulation [19].

To investigate further how *CARD14* mutations induce psoriasis, we and others have generated knock-in mice that constitutively express psoriasis-associated CARD14 mutations that alter or delete the E138 residue [20–22]. Collectively, these mice indicate that CARD14 E138 alterations can induce psoriasisform dermatitis, characterized by keratinocyte proliferation, epidermal acanthosis, hyperkeratosis, immune cell infiltration and increased expression of a number of cytokine/chemokine transcripts up-regulated in psoriasis. The skin inflammation that develops is partially dependent on the cytokines IL17A and IL23 [20,22]. We have also generated a new knock-in mouse allele *Card14*^LSL-E138A^ that allows conditional expression of CARD14^E138A^ in adult mice. Tamoxifen-induced expression of CARD14^E138A^ in *Card14*^LSL-E138A^ mice rapidly induces TNF-dependent psoriasiform skin inflammation, which is independent of adaptive immune cells and due to signalling in keratinocytes, which express high levels of CARD14 [5,23]. CARD14-induced psoriatic dermatitis is also dependent on MALT1 paracaspase activity in keratinocytes [19,24].

Although it is known that psoriasis-associated CARD14 mutations induce the association of CARD14 with BCL10 and MALT1, knowledge about the signalling proteins and pathways involved in CARD14 signalling is rather limited [25]. In the current study, a multi-protein CARD14 signalling complex that is induced by the psoriasis-associated CARD14^E138A^ variant, but not by wild type (WT) CARD14, was characterized in keratinocytes. Several key component proteins of the complex that control CARD14^E138A^ pathogenic signalling were identified. The E3 ubiquitin ligases HOIP [26] and TRAF6 [27] were found to play essential roles in CARD14^E138A^ activation of NF-κB and MAP kinase pathways. Earlier overexpression experiments in HEK293 cells have also suggested a role for TRAF6 in signalling via WT CARD14 [22]. CARD14^E138A^ signalling was found to be negatively regulated by TRAF2-dependent recruitment to the signalosome of cIAP1, another E3 ligase [28] and by CARD14^E138A^ association with the ubiquitin binding proteins A20 (TNFAIP3) and ABIN1 (TNIP1) [29–31]. Each of these negative regulators controlled CARD14^E138A^ expression levels at a post-transcriptional level. In addition, the AP-2 adaptor complex, which regulates endocytic trafficking [32], was identified as a novel component of the CARD14^E138A^ signalosome. Consistent with this, CARD14^E138A^ was shown to be targeted to early endosomes, while WT CARD14 was distributed diffusely in the cytoplasm. AP2 was dispensable for CARD14^E138A^ activation of NF-κB but was required for activation of endolysosomal mTOR complex 1 (mTORC1), a novel downstream target of CARD14 signalling discovered in this study. CARD14^E138A^ stimulated keratinocyte metabolism via mTOR kinase activation. Furthermore, mTORC1 activity was shown to promote CARD14^E138A^ induction of keratinocyte proliferation and epidermal acanthosis in mice. These novel findings suggest that activation of mTORC1 by CARD14 may play an important role in controlling keratinocyte proliferation in psoriasis.

## Results

### Characterization of the CARD14^E138A^ signalosome

The activation of NF-κB by gain-of-function psoriasis-associated CARD14 mutants involves CARD14 forming a complex with BCL10 and MALT1 [17,18]. However, the precise mechanism by which this CARD14-nucleated CBM complex activates NF-κB and is regulated have not been established. It was not possible to obtain primary keratinocytes from patients with psoriasis-associated *CARD14* mutations to explore this question due their rarity. Consequently, to investigate CBM-mediated signalling in detail, we used a model system in which CARD14^E138A^-3xFLAG was inducibly expressed in HaCaT-TR human keratinocytes. HaCaT-TR cells inducibly expressing CARD14^WT^-3xFLAG were used as controls. *CARD14^E138A^* was chosen as this mutation leads to higher levels of NF-κB activation than other mutations [5,16] and mouse studies have demonstrated that *Card14* E138 mutations induce a psoriasiform dermatitis that is strikingly similar to human psoriatic skin [19–23].

**Figure 1.**
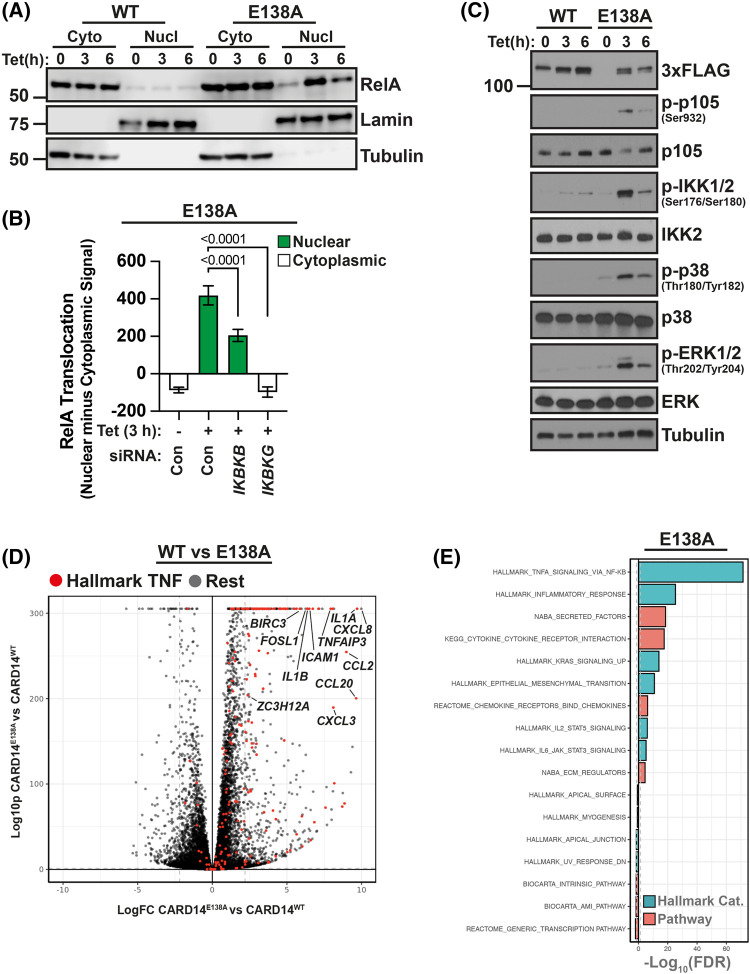
CARD14^E138A^ activates a transcriptional response similar to TNF stimulation in HaCaT-TR keratinocytes. (**A**) CARD14^WT^-3xFLAG or CARD14^E138A^-3xFLAG expression was induced in HaCaT-TR cells with tetracycline (Tet; 3 h). Cytoplasmic and nuclear fractions were immunoblotted for the indicated antigens. (**B**) CARD14E138A-3xFLAG HaCaT-TR cells, incubated with siRNAs targeting IKK2 or NEMO or control non-targeting siRNAs, were cultured with tetracycline (Tet; 3 h). RelA nuclear translocation was determined by confocal microscopy assay, with positive values indicating increased nuclear RelA and negative values indicating cytoplasmic RelA (mean ± SD). Two-tailed unpaired *t*-test. Representative of two independent experiments. Anti-FLAG staining demonstrated that CARD14E138A-3xFLAG expression was not altered by IKK2 or NEMO siRNA knockdown. (**C**) Lysates of CARD14^WT/E138A^-3xFLAG HaCaT-TR cells, incubated with tetracycline (Tet) for the times shown, were immunoblotted for the indicated antigens. (**D**) Volcano plot of gene expression in CARD14^WT^-3xFLAG versus CARD14^E138A^-3xFLAG HaCaT-TR cells, as determined by RNA sequencing. Data from 6 h timepoint (+ tetracycline), with biologically significant and strongly up-regulated genes are highlighted. Red circles indicate genes shared with the hallmark TNF/NF-κB response. (**E**) −Log_10_(FDR) comparison of CARD14^E138A^-3xFLAG-induced differential gene expression with indicated hallmark and pathway genesets.

In initial experiments, we validated the known NF-κB and MAP kinase activating functions of CARD14^E138A^ in our inducible HaCaT-TR cellular model. The effects of CARD14^E138A^-3xFLAG and CARD14^WT^-3xFLAG expression on NF-κB activation were determined by sub-cellular fractionation and immunoblotting. CARD14^E138A^-3xFLAG induced RelA translocation from the cytoplasm to the nucleus, while CARD14^WT^-3xFLAG did not induce RelA nuclear localization (Figure 1A). Confocal imaging of RelA localization confirmed these findings (Supplementary Figure S1A). SiRNA knockdown showed that CARD14^E138A^-3xFLAG induced RelA nuclear translocation was dependent on the IKK complex subunits IKK2 and NEMO (Figure 1B), as expected [18]. Consistent with these results, CARD14^E138A^-3xFLAG activated IKK2 and induced IKK2 phosphorylation of NF-κB1 p105 (p105) (Figure 1C). CARD14^E138A^-3xFLAG also induced activation of ERK1/2 and p38α MAP kinases, as shown previously [18]. In contrast, CARD14^WT^-3xFLAG did not activate IKK2, promote phosphorylation of p105 or activate MAP kinases. Detection with anti-FLAG confirmed inducible expression of CARD14^E138A^-3xFLAG and CARD14^WT^-3xFLAG (Figure 1C).

Next, gene expression in HaCaT keratinocytes was determined in a time course experiment by RNA sequencing. Principal component analysis showed that expression of CARD14^WT^-3xFLAG did not result in notable changes in overall gene expression at any of the time points tested (3, 6 and 9 h; Supplementary Figure S1B). In contrast, expression of CARD14^E138A^-3xFLAG induced clear changes in gene expression, with more pronounced differences as the cell culture period was increased (Supplementary Figure S1B). CARD14^E138A^-3xFLAG changed the expression of multiple genes compared with CARD14^WT^-3xFLAG (Figure 1D). Gene set enrichment analysis (GSEA) of the differentially expressed genes in CARD14^E138A^-3xFLAG cells revealed that the hallmark ‘*TNF signalling* via *NF-κB*’ was the most significantly enriched gene set (Figure 1E and Supplementary Figure S1C), similar to the skin transcriptome of *Card14*^LSL-E138A/+^ *Rosa26^CreERT2/+^* mice following tamoxifen induction of CARD14^E138A^ [23]. Plots of normalized counts from the RNAseq analyses showed that CARD14^WT^-3xFLAG did not change the mRNA abundance of key psoriasis-associated genes, in contrast with CARD14^E138A^-3xFLAG which strongly induced mRNA expression of each gene (Supplementary Figure S1D). The inducible HaCaT system therefore was able to monitor the specific signalling effects of the activating *CARD14^E138A^* mutation (rather than CARD14 overexpression) and reflected physiological CARD14^E138A^ activation of gene expression in primary keratinocytes. Therefore, we utilized this model to further characterize CARD14 biology.

**Figure 2.**
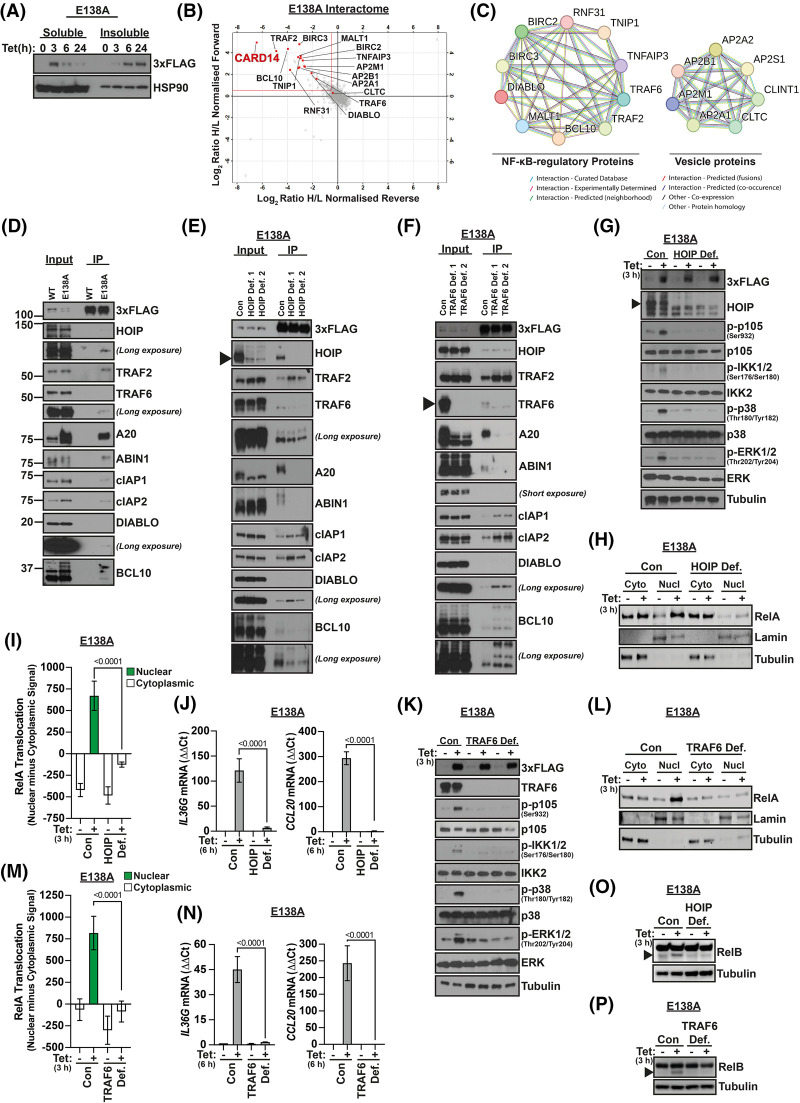
Characterization of the CARD14^E138A^ signalosome. (**A**) CARD14^E138A^-3xFLAG HaCaT-TR cells were incubated for the indicated times with tetracycline (Tet). RIPA cell lysates were separated into soluble and insoluble fractions, resolved by SDS–PAGE and immunoblotted for 3xFLAG and HSP90 (loading control). (**B**) CARD14^E138A^-3xFLAG HaCaT-TR cell lysates were mixed with lysates of HaCaT-TR parental control cell line to create forward and reverse SILAC conditions. Eluates of 3xFLAG pull downs from these mixtures were analyzed by MS. Scatter plot of Log_2_ ratio of heavy (H) versus light (L) normalized peptide intensities for forward and reverse SILAC conditions. CARD14^E138A^-3xFLAG-associated proteins found in both experimental conditions are located in upper left quadrant. Positions of CARD14 and signalosome components are indicated. Log_2_ ratio 0.5 cut-off is shown with a red line. (**C**) Known and computationally derived STRING protein-protein association network for CARD14^E138A^-3xFLAG [33]. (**D**) Lysates of CARD14^WT^-3xFLAG HaCaT-TR (WT) and CARD14^E138A^-3xFLAG (E138A) HaCaT-TR cells, cultured for tetracycline (Tet; 3 h), were immunoprecipitated with anti-FLAG beads. Immunoprecipitates (IP) and input lysates were immunoblotted for the indicated antigens. Molecular mass (kDa) are shown. (**E**) Two monoclonal CARD14^E138A^-3xFLAG HaCaT-TR (Cas9) cell lines deficient in HOIP (HOIP Def. 1, 2) and control non-targeted cells (Con) were cultured with tetracycline (Tet; 3 h). Cell lysates were immunoprecipitated with anti-FLAG. Immunoprecipitates (IP) and input lysates were immunoblotted for the indicated antigens. (**F**) TRAF6-deficient CARD14^E138A^-3xFLAG HaCaT-TR (Cas9) cell lines (TRAF6 Def. 1, 2) and control non-targeted cells (Con) were analyzed as in Figure 2E. (**G**) CARD14^E138A^-3xFLAG HaCaT-TR (Cas9) cell lines deficient in HOIP (HOIP Def. 1, 2) and control non-targeted cells (Con) were cultured ± tetracycline (Tet; 3 h). Lysates were immunoblotted for the indicated antigens. (**H**) Cytoplasmic and nuclear fractions from control (Con) and HOIP-deficient (HOIP Def.) CARD14^E138A^-3xFLAG HaCaT-TR (Cas9) cells ± tetracycline (Tet; 3 h) were immunoblotted. Lamin (nuclear) and tubulin (cytoplasm) blotting confirmed effective fractionation. (**I**) RelA nuclear localization in control non-targeted (Con) and HOIP-deficient (HOIP Def.) CARD14^E138A^-3xFLAG HaCaT-TR (Cas9) cells cultured ± tetracycline (Tet; 3 h) was determined by confocal microscopy. Positive values indicate increased nuclear RelA and negative values indicating cytoplasmic RelA (mean ± SD). Two-tailed unpaired *t*-test. (**J**) CARD14^E138A^-3xFLAG HaCaT-TR (Cas9) cell lines deficient in HOIP (HOIP Def.) and control non-targeted cells (Con) were cultured ± tetracycline (Tet; 6 h). *IL36G* and *CCL20* mRNA expression was determined by qRT-PCR (mean ± SD). Two-tailed unpaired *t*-test. (**K**) TRAF6-deficient CARD14^E138A^-3xFLAG HaCaT-TR (Cas9) cells (TRAF6 Def.) and control non-targeted cells (Con) were analyzed as in Figure 2G. (**L**) TRAF6-deficient CARD14^E138A^-3xFLAG HaCaT-TR (Cas9) cells (TRAF6 Def.) and control non-targeted cells (Con) were analyzed as in Figure 2H. (**M**) TRAF6-deficient CARD14^E138A^-3xFLAG HaCaT-TR (Cas9) cells (TRAF6 Def.) and control non-targeted cells (Con) were analyzed as in Figure 2I. (**N**) TRAF6-deficient CARD14^E138A^-3xFLAG HaCaT-TR (Cas9) cells (TRAF6 Def.) and control non-targeted cells (Con) were analyzed as in Figure 2J. (**O**) CARD14^E138A^-3xFLAG HaCaT-TR (Cas9) cell lines deficient in HOIP (HOIP Def.) and control non-targeted cells (Con) were cultured ± tetracycline (Tet; 3 h). Lysates were immunoblotted for RelB. Arrowhead shows position of proteolysed RelB band. (**P**) TRAF6-deficient CARD14^E138A^-3xFLAG HaCaT-TR (Cas9) cells (TRAF6 Def.) and control non-targeted cells (Con) were analyzed as in Figure 2O.

Signalling by CARD14^E138A^ has been linked to formation of large detergent insoluble cytoplasmic complexes [20]. At 3 h post-tetracycline induction CARD14^E138A^-3xFLAG was predominantly detergent soluble and only after 6 h culture formed insoluble complexes (Figure 2A). In contrast, CARD14^WT^-3xFLAG remained soluble for up to 6 h post-tetracycline induction (Supplementary Figure S1E), retaining a diffuse cytosolic distribution up to 24 h in culture in contrast with CARD14^E138A^-3xFLAG which was all aggregated by 24 h (Supplementary Figure S1F). These results demonstrated that CARD14^E138A^-3xFLAG formation of detergent insoluble complexes was associated with reduced activation of downstream signalling pathways (Figure 1C and Supplementary Figure S1G). Maximal signalling occurred when CARD14^E138A^ was soluble and indicated that characterization of soluble CARD14^E138A^ complexes was important to determine mechanisms of signalling.

To investigate in more detail how CARD14^E138A^ signals, the composition of soluble CARD14^E138A^-3xFLAG complexes was determined by mass spectrometry (MS). CARD14^E138A^-3xFLAG was immunoprecipitated with anti-FLAG from lysates of CARD14^E138A^-3xFLAG HaCaT-TR cells generated after 3 h of tetracycline-induction. To control for variations in sample runs, stable-isotope labelling by amino acids in cell culture (SILAC) was used to discriminate anti-FLAG pulldowns from CARD14^E138A^-3xFLAG HaCaT-TR cells with anti-FLAG control pulldowns from HaCaT-TR (control) cells (Supplementary Figure S1H).

In line with our earlier findings [17,18], CARD14^E138A^-3xFLAG was specifically associated with BCL10 and MALT1 (Figure 2B). However, a number of other proteins linked to NF-κB signalling were also identified suggesting a greater complexity of regulation in CARD14^E138A^ signal transduction (Figure 2C). These included the E3 ubiquitin ligases TRAF6 and cIAP1/2 (BIRC2/3) as well as HOIP (RNF31), the catalytic subunit of the linear ubiquitin chain assembly complex (LUBAC) [26]. In addition, CARD14^E138A^-3xFLAG pulldowns contained ABIN1 (TNIP1) and A20 (TNFAIP3), the ubiquitin binding proteins which are encoded by genes that reside in loci identified through psoriasis GWAS [34]. Immunoprecipitation and immunoblotting confirmed interaction of these proteins with CARD14^E138A^-3xFLAG (Figure 2D). Critically, none of these proteins interacted with CARD14^WT^-3xFLAG (Figure 2D), consistent with MS analysis of CARD14^WT^-3xFLAG pulldowns that identified few associated proteins (Supplementary Figure S1I). Previous overexpression experiments in HEK293 cells have indicated that CARD14^WT^ can associate with TRAF6 [22], but this association was not detected in the inducible HaCaT-TR cell system. A20 was expressed at much lower levels in CARD14^WT^-3xFLAG cells compared with CARD14^E138A^-3xFLAG cells, consistent with its known transcriptional activation by NF-κB [35]. Since expression of CARD14^WT^-3xFLAG in HaCaT-TR cells did not activate downstream signalling pathways, these data suggested that proteins associated with CARD14^E138A^-3xFLAG were involved in active CARD14^E138A^ signalling.

### CARD14^E138A^ signalling is positively and negatively regulated by signalosome components

Our results demonstrated that the *CARD14*^E138A^ alteration induced the formation of a complex CARD14 signalosome containing a number of signalling proteins in addition to BCL10 and MALT1. Several of these have previously already been shown to have roles in CARD11 signalling in T and B cells, suggesting that CARD14 shared signalling mechanisms with CARD11 [12].

To investigate the requirements of CARD14^E138A^-3xFLAG-associated proteins for complex assembly and signalling, monoclonal cell lines deficient in specific signalosome components were generated with Cas9-stable CARD14^E138A^-3xFLAG HaCaT-TR cells and CRISPR-mediated gene editing [36]. Immunoblotting confirmed successful generation of cell lines deficient in each of the targeted proteins. To generate cIAP1-deficient cells, the small molecule inhibitor of IAP birinapant (TL32711) was used to induce cIAP1 auto-degradation [37].

CARD14^E138A^-3xFLAG expression was induced with tetracycline for 3 h in control and two separately generated HOIP knockout cell lines, which expressed similar levels of CARD14^E138A^-3xFLAG to WT parental cells after tetracycline induction (Figure 2E). Signalosome composition was determined by anti-FLAG immunoprecipitation and immunoblotting. HOIP expression was required for association of CARD14^E138A^-3xFLAG with A20 and ABIN1, which was in line with their known ability to bind M1 polyubiquitin chains synthesized by HOIP [30,31] (Figure 2E). TRAF6 deficiency also substantially reduced co-immunoprecipitation of A20 and ABIN-1 with CARD14^E138A^-3xFLAG, with minimal effects on steady-state CARD14^E138A^-3xFLAG levels after tetracycline induction (Figure 2F). The reduction in A20 association with CARD14^E138A^-3xFLAG in both HOIP- and TRAF6-deficient cells was due, in part, to decreased A20 protein levels, consistent with its known transcriptional regulation by NF-κB [35]. HOIP and TRAF6 interacted with CARD14^E138A^-3xFLAG independently of one another. The association of TRAF2 and cIAP1/2 with CARD14^E138A^-3xFLAG did not require HOIP or TRAF6 expression and was even increased in the absence of TRAF6 or HOIP. Noteworthy, BCL10 binding to CARD14^E138A^-3xFLAG was reduced in the absence of HOIP, whilst increased in the absence of TRAF6, further illustrating the complex role of different types of polyubiquitination in the assembly and/or stability of the CARD14 signalosome.

ABIN1 was originally identified as an A20 binding protein and has been proposed to negatively regulate TNF- and Toll-like receptor (TLR)-induced NF-κB signalling in concert with A20 [38]. However, A20 and ABIN1 could associate with CARD14^E138A^-3xFLAG independently of one another (Supplementary Figure S2A,B). Both proteins also seemed to negatively regulate each other's expression levels, possibly reflecting their NF-κB-dependent transcription. Neither ABIN1 nor A20 were required for association of HOIP, TRAF6, cIAP1/2 or TRAF2 with CARD14^E138A^-3xFLAG, although cIAP1 binding was strongly reduced in the absence of ABIN1 (Supplementary Figure S2B). TRAF2-deficiency reduced the association of cIAP1/2 and the cIAP binding protein DIABLO with CARD14^E138A^-3xFLAG, without affecting HOIP, TRAF6, A20 or ABIN1 binding (Supplementary Figure S2C). cIAP1 and cIAP2 interacted with CARD14^E138A^-3xFLAG independently of one another but DIABLO association was cIAP1 dependent (Supplementary Figure S2D,E). cIAP1/2 were not required for CARD14^E138A^-3xFLAG association with HOIP, TRAF6, TRAF2, A20 and ABIN1. A summary of the interaction network of CARD14^E138A^ signalosome components is shown in Supplementary Figure S2F.

Deficiency of the E3 ligases HOIP and TRAF6 blocked the activation of downstream IKK2 and MAP kinase signalling pathways, the nuclear translocation of RelA, and induction of proinflammatory gene expression by CARD14^E138A^-3xFLAG (Figure 2G–N). The block in IKK2/NF-κB activation in the absence of HOIP and TRAF6 explained the reduction in expression of A20, which is encoded by an NF-κB regulated gene [39]. Activation of MALT1 paracaspase activity following CARD14^E138A^ expression [40], as monitored by RelB proteolysis, was also HOIP- and TRAF6-dependent (Figure 2O,P).

In contrast, the absence of A20 augmented IKK2 activation and p105 phosphorylation following CARD14^E138A^-3xFLAG induction, resulting in increases of both RelA nuclear translocation and pro-inflammatory gene expression (Supplementary Figure S3A–C). A20 deficiency also resulted in fractional increases in p38α and ERK1/2 activation, although the extents of these changes were somewhat variable between experiments (Supplementary Figure S3A). siRNA knockdown in CARD14^E138A^-3xFLAG HaCaT-TR cells confirmed that A20 deficiency increased CARD14^E138A^-3xFLAG induction of p105 and MAPK phosphorylation (Supplementary Figure S3E). Similar effects were observed in the absence of ABIN1 (Supplementary Figure S3F–H). The detected increases in signalling in the absence of A20 or ABIN1 correlated with elevated levels of CARD14^E138A^-3xFLAG in cell lysates (Supplementary Figure S3A,E,F), although tetracycline-induced *CARD14^E138A^-3xFLAG* mRNA levels were not significantly altered by the absence of A20 or ABIN1 (Supplementary Figure S3D). TRAF2 deficiency also increased CARD14^E138A^-3xFLAG expression and activation of downstream signalling, without significantly altering *CARD14^E138A^ 3xFLAG* mRNA levels (Supplementary Figure S3D,I–K). cIAP2-deficiency did not change activation of IKK2 and MAP kinases or significantly modify RelA nuclear translocation (Supplementary Figure S3L,N). In contrast, cIAP1 depletion with birinapant increased CARD14^E138A^-3xFLAG levels and activation of downstream signalling pathways (Supplementary Figure S3M). Depletion of cIAP1 in cIAP2-deficient cells did not cause significantly greater levels of RelA translocation when compared with cIAP1 depletion alone (Supplementary Figure S3N).

Overall these data show that, similar to CARD11 signalling [12], CARD14^E138A^ signalling was controlled by the interaction of CARD14^E138A^ with multiple ubiquitin-regulatory proteins. HOIP and TRAF6 E3 ubiquitin ligases were essential for CARD14^E138A^-induced activation of downstream pathways and were also necessary for the independent recruitment of A20 and ABIN1 ubiquitin binding proteins that negatively regulated CARD14^E138A^ signalling. The inhibitory effects of A20 and ABIN1 on CARD14^E138A^ signalling were consistent with our earlier overexpression experiments in HEK293 cells [17]. TRAF2 also inhibited CARD14^E138A^ signalling, possibly via recruitment of the negative regulator cIAP1 and associated DIABLO. Each identified negative regulators appeared to modulate CARD14^E138A^ signalling by decreasing CARD14^E138A^ expression post-transcriptionally, suggesting that the stability of CARD14^E138A^ may be regulated by associated component proteins in the signalosome. Supplementary Figure S3O summarizes the roles of signalosome components in CARD14^E138A^ signalling.

### HOIP and TRAF6 promote BCL10 ubiquitination and activation of TAK1 by CARD14^E138A^

Our previous work has demonstrated that the *CARD14*^E138A^ alteration induces the formation of a CARD14^E138A^-BCL10-MALT1 complex that activates NF-κB [17,18]. To investigate the roles of BCL10 and MALT1 in formation of the expanded CARD14^E138A^ signalosome, BCL10 and MALT1 knockout CARD14^E138A^-3xFLAG HaCaT-TR cell lines were generated. The absence of either BCL10 or MALT1 significantly reduced levels of CARD14^E138A^-3xFLAG (3xFLAG blot) (Supplementary Figure S4A,B). siRNA knockdown experiments confirmed that BCL10 and MALT1 were required to maintain levels of CARD14^E138A^-3xFLAG but were not needed to maintain CARD14^WT^-3xFLAG levels (Supplementary Figure S4C). These results suggested that the absence of either BCL10 or MALT1 may have destabilized CARD14^E138A^-3xFLAG or reduced CARD14^E138A^-3xFLAG mRNA levels. Consequently, we were not able to determine how BCL10 and MALT1 affected the assembly of the CARD14^E138A^-3xFLAG signalosome.

**Figure 3.**
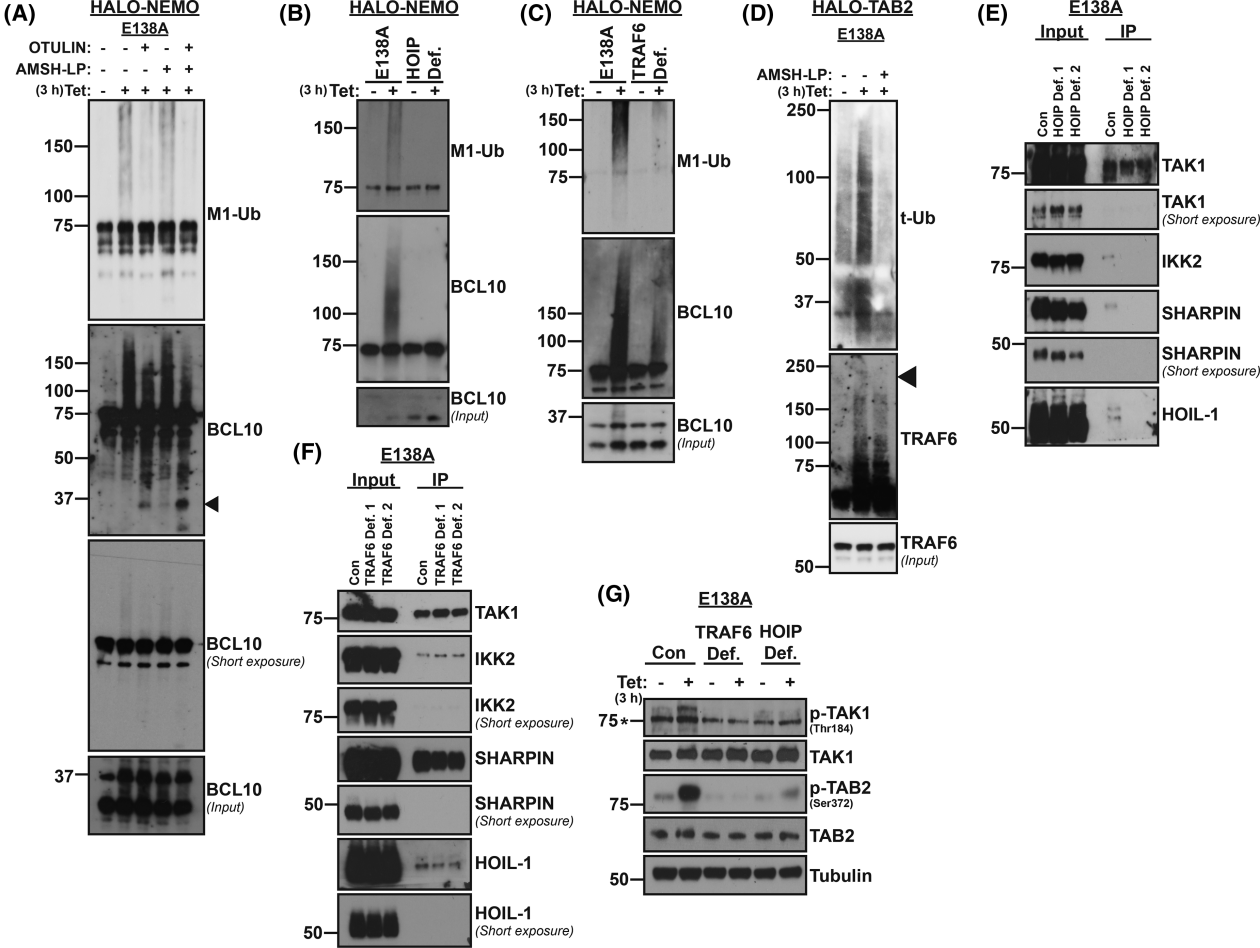
CARD14^E138A^ induces HOIP- and TRAF6-dependent ubiquitination of BCL10. (**A**) HALO-NEMO pull downs were performed with lysates from CARD14^E138A^ 3xFLAG HaCaT-TR cells (E138A) incubated ± tetracycline (Tet; 3 h). HALO-NEMO resin was then treated with the M1-Ub and/or K63-Ub specific DUBs, OTULIN (1 μM) and AMSH-LP (0.2 μM), respectively. Control pulldowns (-) were left untreated. Pulldowns and input lysates were immunoblotted for the indicated antigens. (**B**) HALO-NEMO pull downs were carried out with HOIP-deficient (HOIP Def.) and control non-targeted (Con) CARD14^E138A^ 3xFLAG HaCaT-TR (Cas9) cells as in E. Pull downs and input lysates were immunoblotted. (**C**) HALO-NEMO pull downs were carried out with TRAF6-deficient (TRAF6 Def.) and control non-targeted (Con) CARD14^E138A^ 3xFLAG HaCaT-TR (Cas9) cells as in **E**. Pull downs and input lysates were immunoblotted. (**D**) HALO-TAB2 (K63-Ub) pull downs were performed with lysates from CARD14^E138A^ 3xFLAG HaCaT-TR cells cultured ± tetracycline (Tet; 3 h). HALO-NEMO resin was then treated with the K63-Ub specific DUB AMSH-LP (+) or left untreated (−). Pulldowns and input lysates were immunoblotted. (**E**) Two monoclonal CARD14^E138A^-3xFLAG HaCaT-TR (Cas9) cell lines deficient in HOIP (1, 2) and control non-targeted cells (C) were cultured with tetracycline (3 h). Lysates were immunoprecipitated with anti-FLAG and immunoprecipitates (IP) immunoblotted for the indicated antigens. (**F**) Two monoclonal CARD14^E138A^-3xFLAG HaCaT-TR (Cas9) cell lines deficient in TRAF6 (1, 2) and control non-targeted cells (C) were cultured with tetracycline (3 h). Lysates were immunoprecipitated with anti-FLAG and immunoprecipitates (IP) immunoblotted for the indicated antigens. (**G**) Lysates from control (Con), TRAF6-deficient (TRAF6 Def.) and HOIP-deficient (HOIP Def.) CARD14^E138A^ 3xFLAG HaCaT-TR (Cas9) cells were immunoblotted for the indicated antigens. Molecular mass (kDa) are shown. All data in this figure are representative of at least two similar independent experiments.

CARD14 interaction with BCL10 is mediated via CARD domains [10]. Since HOIP was required for maximal BCL10 interaction with CARD14^E138A^-3xFLAG (Figure 2E), the possibility that BCL10 was modified with linear M1 polyubiquitin (Ub) chains was investigated. CARD14^E138A^-3xFLAG expression was induced for 3 h with tetracycline, and cell lysates were incubated with beads linked to HALO-NEMO (binds M1 >> K63 Ub chains) or HALO-TAB2 (two copies of the NZF domain of TAB2 that binds to K63 Ub chains) recombinant proteins to isolate proteins covalently modified with M1-Ub and/or K63-Ub [41]. Induction of CARD14^E138A^-3xFLAG promoted the formation of M1-Ub oligomer chains in cell lysates (Figure 3A). BCL10 was detected in HALO-NEMO pulldowns, with high molecular mass forms induced by CARD14^E138A^-3xFLAG expression (Figure 3A). The high molecular mass forms of BCL10 were due to M1 ubiquitination as incubation with the M1-specific DUB OTULIN [42] reduced the size of the chains attached to BCL10. Consistently, CARD14^E138A^-3xFLAG-induced M1-linked ubiquitination of BCL10 was dependent on HOIP expression (Figure 3B).

The role of TRAF6 in CARD14^E138A^-3xFLAG signalosome ubiquitination was also investigated. Although TRAF6 was not detected in HALO-NEMO pulldowns, TRAF6 expression was required for CARD14^E138A^-3xFLAG-induced M1 ubiquitination in cell lysates and of BCL10 (Figure 3C). HALO-TAB2 pulldowns demonstrated that CARD14^E138A^-3xFLAG expression induced the assembly of K63-Ub chains in cell lysates and linked to TRAF6 (Figure 3D). TRAF6 is similarly required for M1 ubiquitination induced by MyD88, which involves the formation of K63/M1-Ub hybrid chains on MyD88 [43]. Treatment with K63-specific DUB AMSH-LP had little effect on high molecular mass forms of BCL10 (Figure 3A). However, the combination of OTULIN plus AMSH-LP resulted in increased levels of fully deubiquitinated BCL10 (28 kDa band indicated with arrow). The requirement for TRAF6 to induce M1 ubiquitination of BCL10 and the increased efficiency of BCL10 deubiquitination with the combination of OTULIN and AMSH-LP suggests that CARD14^E138A^ signaling also involved the attachment of K63/M1-Ub hybrid chains to BCL10.

In TLR and IL-1R pathways, the cooperative functions of TRAF6 and HOIP, as a component of LUBAC, promote the downstream activation of NF-κB by the IKK complex. A model has been proposed in which K63-linked chains catalyzed by TRAF6 recruit the TAB2-TAK1 complex that functions upstream of the IKK complex, which is itself recruited by linear ubiquitin chains produced by LUBAC [43]. The formation of hybrid K63/M1 linked Ub chains may facilitate this process by simultaneously recruiting TAK1 and IKK to the same upstream regulator. The roles of HOIP and TRAF6 function in CARD14^E138A^ interaction with the TAB2-TAK1 and IKK complexes were investigated. Initial experiments confirmed that the other components of LUBAC, SHARPIN and HOIL-1 [26], were recruited to the CARD14^E138A^-3xFLAG signalosome in a HOIP-dependent fashion (Figure 3E). Association of the catalytic IKK2 subunit of the IKK complex with CARD14^E138A^-3xFLAG was also HOIP-dependent, while TAK1 association was not altered by HOIP deficiency. Associations of SHARPIN and HOIL-1 with CARD14^E138A^-3xFLAG were also TRAF6-independent (Figure 3F), similar to HOIP (Figure 2F). IKK2 and TAK1 recruitment were also unaffected by TRAF6 deficiency (Figure 3F). However, CARD14^E138A^ induced phosphorylation of TAB2 and TAK1 was strongly dependent on TRAF6 and HOIP expression (Figure 3G). TAB2 siRNA knockdown demonstrated that CARD14^E138A^-3xFLAG-induced phosphorylation of p105 by IKK was dependent on the TAB2 component of the TAK1 complex (Supplementary Figure S4D).

These results indicate that HOIP catalyzed M1 ubiquitination of BCL10, which may have stabilized its interaction with CARD14^E138A^, in addition to promoting the recruitment of the IKK complex to the CARD14^E138A^ signalosome. Although TAK1 association with CARD14^E138A^ was HOIP and TRAF6 independent, TRAF6- and HOIP-mediated ubiquitination was required for TAK1 activation. Consequently, activation of NF-κB by CARD14^E138A^ was dependent on co-ordinated ubiquitination mediated by HOIP and TRAF6.

### CARD14^E138A^ localizes to early endosomes associated with the microtubule network

**Figure 4.**
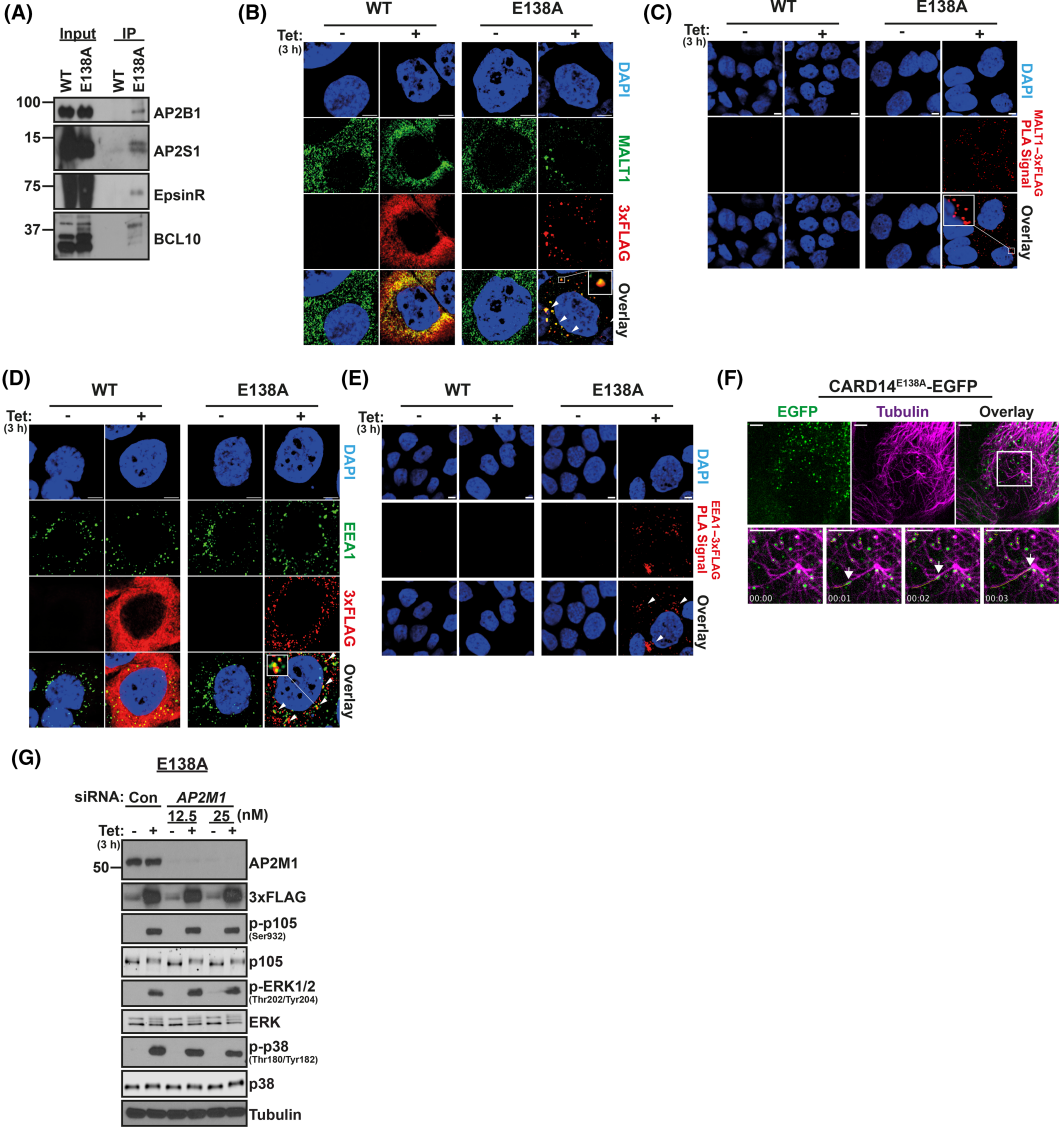
CARD14^E138A^ is associated with early endosomes. (**A**) Lysates of CARD14^WT^-3xFLAG HaCaT-TR (WT) and CARD14^E138A^-3xFLAG HaCaT-TR cells (E138A), cultured for 3 h with tetracycline, were subjected to immunoprecipitation with anti-FLAG beads. Immunoprecipitates (IP) and input lysates were immunoblotted for the indicated antigens. Molecular mass (kDa) are shown. (**B**) Confocal microscopy imaging of anti-FLAG and anti-MALT1 localization in CARD14^WT^-3xFLAG HaCaT-TR (WT) and CARD14^E138A^-3xFLAG (E138A) HaCaT-TR cells ± tetracycline induction (Tet; 3 h). Nuclei were stained with DAPI. (**C**) Proximity ligations assays determined whether CARD14^WT^-3xFLAG (WT) and CARD14^E138A^-3xFLAG (E138A) co-localized with MALT1 (by anti-FLAG and anti-MALT1 staining) in HaCaT-TR cells ± tetracyline induction (Tet; 3 h). Overlay box (E138A + Tet) shows blow up of indicated region. (**D**) Confocal microscopy imaging of anti-FLAG and anti-EEA1 localization in CARD14^WT^-3xFLAG HaCaT-TR (WT) and CARD14^E138A^-3xFLAG (E138A) HaCaT-TR cells ± tetracycline induction (Tet; 3 h). Nuclei were stained with DAPI. Arrowheads show regions of coincident staining. Overlay box (E138A + Tet) shows blow up of indicated region. (**E**) Proximity ligations assays determined whether CARD14^WT^-3xFLAG (WT) and CARD14^E138A^-3xFLAG (E138A) were in close proximity with EEA1 (by anti-FLAG and anti-EEA1 staining) in HaCaT-TR cells ± tetracycline induction (Tet; 3 h). (**F**) Confocal microscopy localization of CARD14^E138A^-EGFP (EGFP) induced by tetracycline (3 h) in live HaCaT-TR cells was determined. Microtubules were fluorescently labelled with SiR-Tubulin (Tubulin). Lines tracking movement of CARD14^E138A^-EGFP foci were created post-acquisition using TrackMate (ImageJ). Time stamp = mm:ss, scale bar = 5 μm. Images are representative of several acquisitions across separate cells and those shown are obtained from Supplementary Video S1. Bottom row of images show time series from region highlighted with box in overlay panel. (**G**) CARD14^E138A^ 3xFLAG HaCaT-TR cells were pre-treated with *AP2M1* or control (con) siRNA. Cell lysates were prepared 3 h after tetracycline induction (Tet; 3 h) and immunoblotted for the indicated antigens. All data in this figure are representative of at least two replicate experiments.

A number of CARD14^E138A^-3xFLAG-interacting proteins discovered by MS are known to be important in vesicular trafficking (Figure 2C). These included all of the subunits of the AP2 complex and EpsinR (CLINT1), which are involved in clathrin-mediated endocytosis [32]. Immunoprecipitation and immunoblotting demonstrated that AP2 (probing for AP2B1 and AP2S1 subunits) and EpsinR interacted with CARD14^E138A^-3xFLAG but not with CARD14^WT^-3xFLAG in HaCaT-TR keratinocytes (Figure 4A). These results raised the possibility that CARD14^E138A^ signalling may be physically linked to endosomes.

High resolution confocal microscopy was used to characterize the intracellular localization of active CARD14^E138A^-3xFLAG complexes at 3 h. This revealed that CARD14^E138A^-3xFLAG staining is associated with small puncta distributed throughout the cytoplasm at peak signalling, suggestive of CARD14^E138A^-3xFLAG localization to endocytic vesicles (Figure 4B). CARD14^E138A^-3xFLAG puncta co-localized with endogenous MALT1 staining, which was confirmed by proximity ligation assays (PLAs) (Figure 4C). These data indicated that CARD14^E138A^-3xFLAG foci were sites of CARD14^E138A^-BCL10-MALT1 assembly. Staining for CARD14^WT^-3xFLAG produced a more diffuse granular cytoplasmic pattern (Figure 4B). PLAs revealed no co-localization of CARD14^WT^-3xFLAG with MALT1 (Figure 4C), consistent with the failure of CARD14^WT^-3xFLAG to form CBM complexes [18] or trigger downstream signalling pathways.

Immunofluorescence staining and confocal microscopy showed that CARD14^E138A^-3xFLAG puncta partially overlapped with the early endosome marker EEA1 (Figure 4D). PLAs indicated that CARD14^E138A^-3xFLAG was in close proximity with EEA1 (Figure 4E). In contrast, PLAs indicated that CARD14^WT^-3xFLAG was not in close proximity with EEA1 (Figure 4D,E). These results showed that *CARD14^E138A^* mutation induced the association of CARD14 signalosomes with early endosomes.

To characterize the distinctive localization of CARD14^E138A^ signalosomes in live cells, stable HaCaT-TR cells were generated that inducibly express CARD14^E138A^-EGFP. At peak signalling for NF-κB activation, CARD14^E138A^-EGFP formed small puncta in the cytoplasm similar to CARD14^E138A^-3xFLAG (Supplementary Figure S4E). By time-lapse microscopy, it was determined that CARD14^E138A^-EGFP was associated with highly motile bodies in the cytoplasm. The vesicle-like localization and nature of signalosome movement suggested that the CARD14^E138A^ signalling complex might assemble along the microtubule network. Live staining for tubulin revealed that CARD14^E138A^-EGFP complexes moved along microtubule paths in keratinocytes (Figure 4F). Tracking measurements of time-lapse data demonstrated that signalosomes migrated along microtubule paths at speeds consistent with that of microtubule motor protein-mediated vesicular transport (Supplementary Figure S4F) [44].

AP2M1 depletion blocks AP2-dependent formation of clathrin-coated pits and receptor-mediated endocytosis [45]. siRNA knockdown of AP2M1 was used to investigate whether CARD14^E138A^ interaction with the AP2 complex was required for activation of NF-κB or MAP kinases. Although AP2M1 was efficiently depleted, CARD14^E138A^-induced phosphorylation of NF-κB1 p105, p38α and ERK1/2 were unaffected (Figure 4G). These data showed that AP2 association was not required for CARD14^E138A^ activation of NF-κB or MAP kinases and raised the possibility that localization to endosomes might be important to couple CARD14^E138A^ to other signalling pathway(s).

### Activation of mTORC1 by CARD14^E138A^

**Figure 5.**
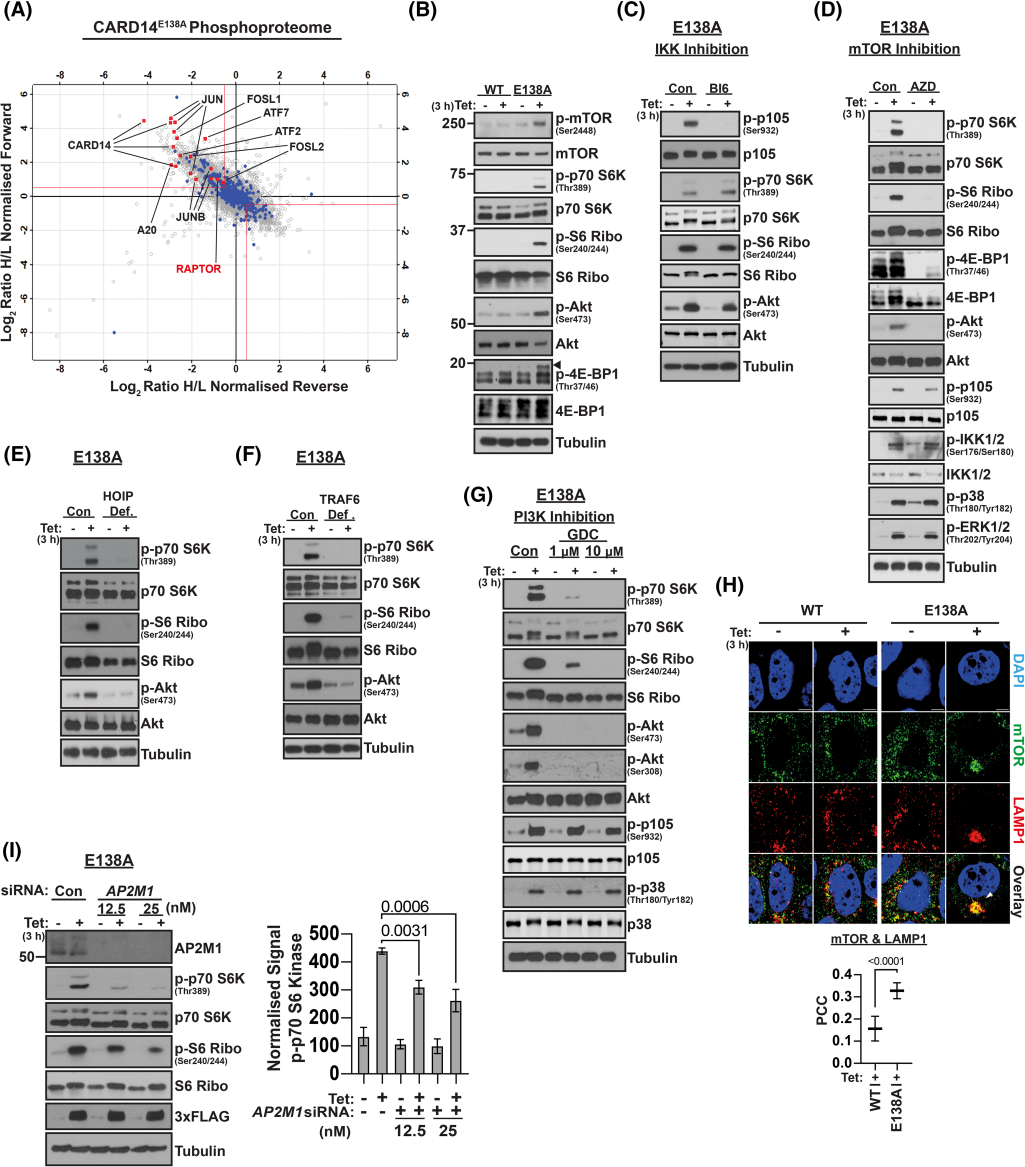
CARD14^E138A^ activates mTOR complex 1 independently of NF-κB activation. (**A**) Parental HaCaT-TR cells or CARD14^E138A^ 3xFLAG HaCaT-TR cells were cultured in heavy or light amino acid SILAC media. Cell lysates were mixed according to Supplementary Figure S1E table and analysed by MS. Scatter plot shows Log_2_ ratio of phosphosite intensities, heavy versus light, normalized from forward and reverse SILAC conditions. Red lines = threshold of Log_2_ ratio (0.5). Hollow grey circle = detected phosphosite. Annotated phosphosites of interest are highlighted by red squares. (**B**) CARD14^WT^-3xFLAG (WT) and CARD14^E138A^-3xFLAG (E138A) were induced with tetracycline (Tet; 3 h) in HaCaT-TR cells. Cell lysates were immunoblotted for the indicated antigens. (**C**) Expression of CARD14^E138A^-3xFLAG was induced with tetracycline (Tet; 3 h) in HaCaT-TR cells (E138A) ± BI605906 (BI6) treatment to block IKK2. Control cells were treated with DMSO vehicle (Con). Cell lysates were immunoblotted. (**D**) Expression of CARD14^E138A^-3xFLAG was induced with tetracycline (Tet; 3 h) in HaCaT-TR cells (E138A) ± AZD8055 (AZD) treatment to block mTOR. Control cells were treated with DMSO vehicle (Con). Cell lysates were immunoblotted. (**E**) HOIP-deficient (HOIP Def.) and control non-targeted (Con) CARD14^E138A^ 3xFLAG HaCaT-TR (Cas9) cells were induced with tetracycline (Tet; 3 h). Cell lysates were immunoblotted. (**F**) TRAF6-deficient (TRAF6 Def.) and control non-targeted (Con) CARD14^E138A^ 3xFLAG HaCaT-TR (Cas9) cells were induced with tetracycline (Tet; 3 h). Cell lysates were immunoblotted. (**G**) Expression of CARD14^E138A^ was induced with tetracycline (Tet; 3 h) in CARD14^E138A^ 3xFLAG HaCaT-TR cells ± GDC-0941 (GDC) treatment (at indicated concentrations) to inhibit PI 3-kinase. Control cells were treated with DMSO vehicle (Con). Cell lysates were immunoblotted. (**H**) Expression of CARD14^WT^-3xFLAG (WT) and CARD14^E138A^-3xFLAG (E138A) was induced with tetracycline (Tet; 3 h) in HaCaT-TR cells. Confocal imaging determined localization of anti-mTOR and anti-LAMP1 staining. Nuclei were stained with DAPI. Pixel data from three independent experiments were used to determine the degree of signal correlation via Pearson's correlation with Costes method in relevant regions of interest. Means (±SD) are presented and tested by unpaired two tailed *t*-test to respective control mean for each cell line. (**I**) CARD14^E138A^ 3xFLAG HaCaT-TR cells were pre-treated with *AP2M1* or control (Con) siRNAs. Cell lysates ± tetracycline induction (Tet; 3 h) were immunoblotted for the indicated antigens. Data for S6 kinase phosphorylation from three independent experiments normalized to tubulin loading control are plotted (means ± SD) and analyzed by one-way ANOVA, with correction for multiple testing.

Our earlier work demonstrated that CARD14^E138A^ promotes inflammation by activation of IKK2 and MAP kinases [17,18]. We reasoned that CARD14^E138A^ interaction with AP2 might be important for activation of other downstream kinases. To investigate this possibility, MS was used to determine how expression of CARD14^E138A^-3xFLAG altered protein phosphorylation in HaCaT-TR keratinocytes (Figure 5A and Supplementary Figure S5A). This analysis demonstrated that CARD14^E138A^ induced Ser381 phosphorylation of A20, which was previously shown to be an IKK2 phosphorylated residue critical for the inhibitory function of A20 [46,47]. Phosphorylation of ERK2 on its activation loop [48] was also induced by CARD14^E138A^-3xFLAG. In addition, CARD14^E138A^-3xFLAG induced phosphorylation of the AP1 transcription factors FRA1 [49], JUN [50] and ATF7 [51] on regulatory sites phosphorylated by MAP kinases. Phosphoproteome analysis therefore confirmed that CARD14^E138A^ activates IKK2 and MAP kinases.

CARD14^E138A^-3xFLAG was also found to promote the phosphorylation of the RAPTOR component of mTORC1 on Ser863, a key event for activation of mTOR [52]. In addition, RICTOR phosphorylation on Thr1135 was induced, which is phosphorylated by mTORC1-activated S6 kinase 1 [53]. Consistent with these findings, immunoblotting demonstrated that CARD14^E138A^-3xFLAG induced mTORC1 to phosphorylate 4E-BP1 and p70 S6 kinase, which then phosphorylated S6 (Figure 5B). CARD14^E138A^-3xFLAG also promoted Ser473 phosphorylation of Akt, which is phosphorylated by mTORC2 [54]. In contrast, CARD14^WT^-3xFLAG expression did not induce mTOR phosphorylation of these proteins. Pharmacological inhibitor experiments in CARD14^E138A^-3xFLAG HaCaT-TR cells, monitoring S6 kinase and S6 phosphorylation, demonstrated that CARD14^E138A^-3xFLAG activation of mTOR was independent of IKK2 activity (BI605906), and the activities of p38α (BIRB796), ERK1/2 (PD0325901) and JNK (JNK-in8) MAP kinases as well as MALT1 paracaspase activity (MLT-748) (Figure 5C and Supplementary Figure S5B–E). Conversely, IKK2 activation and activation of ERK1/2 and p38α MAP kinases by CARD14^E138A^-3xFLAG were not blocked by AZD8055 inhibition of mTOR activity (Figure 5D) or rapamycin inhibition of mTORC1 (Supplementary Figure S5F). Furthermore, CARD14^E138A^-3xFLAG induction of pro-inflammatory gene expression was not altered by AZD8055 (Supplementary Figure S6A). However, analysis of knockout HaCaT-TR cells indicated that CARD14^E138A^-3xFLAG activation of mTOR and phosphorylation of its downstream target S6 kinase required HOIP and TRAF6 (Figure 5E,F), confirming that specific signalosome components were important for mTORC1 pathway activation.

mTORC1 is negatively regulated by the tuberous sclerosis 1 (TSC1)-TSC2 complex, which functions as GTPase-activating protein for Rheb GTPase [55]. The TSC1/TSC2 complex is phosphorylated and inactivated by the protein kinase Akt, which itself is activated by PI 3-kinase [56]. CARD14^E138A^-3xFLAG expression induced the phosphorylation of a key activating site on Akt, Ser473 [56] in HaCaT-TR cells (Figure 5B) and increased TSC2 phosphorylation on an Akt target site, Thr1462 (Supplementary Figure S5G) [57]. MK-2206, an Akt inhibitor, blocked TSC2 Thr1462 phosphorylation as expected and decreased S6 kinase and S6 phosphorylation induced by CARD14^E138A^-mediated activation of mTOR (Supplementary Figure S5G). GDC-0941, a pan-specific PI 3-kinase inhibitor, also blocked CARD14^E138A^-induced Akt Ser473/Ser308 phosphorylation and mTOR phosphorylation of S6 kinase and S6 (Figure 5G). However, GDC-0941 did not affect CARD14^E138A^ activation of IKK2 or p38α and ERK1/2 MAP kinases. These experiments demonstrated that CARD14^E138A^ induced PI 3-kinase-dependent activation of Akt and that CARD14^E138A^-mediated activation of mTORC1, but not IKK2/NF-κB activation, was dependent on PI 3-kinase signalling.

mTORC1 is localized on late endosomes together with its upstream activator the RAGULATOR complex [58,59]. Consistent with this, confocal microscopy indicated that co-localization of mTOR with the late endosome marker LAMP1 was significantly increased by CARD14^E138A^-3xFLAG expression compared with CARD14^WT^-3xFLAG (Figure 5H). Clathrin and AP2 regulate lysosome homeostasis [60] raising the possibility that CARD14^E138A^ interaction with AP2 might be important for activation of mTORC1 on endolysosomes. To investigate this possibility, AP2 function was inhibited by *AP2M1* siRNA knockdown. In contrast with the NF-κB and MAP kinase pathways (Figure 4G), AP2M1 deficiency substantially reduced CARD14^E138A^ activation of mTORC1 and phosphorylation of S6 kinase (Figure 5I). These results indicated that interaction with AP2 coupled the endosomal CARD14^E138A^ signalosome to the activation of mTORC1.

Together the results in this section identified mTORC1 as a novel downstream signalling target of CARD14^E138A^, activated independently of IKK/NF-κB and MAP kinases. CARD14^E138A^ stimulation of mTORC1 required HOIP and TRAF6, similar to IKK/NF-κB, but was specifically dependent on AP2 function (AP2M1 knockdown) and PI 3-kinase activity.

### CARD11^L232LI^ activates mTORC1 independently of AP2

Our earlier analyses revealed similarities between CARD14^E138A^ signalling mechanisms involved in activation of IKK/NF-κB in keratinocytes with those utilized by CARD11 in lymphocytes [12]. However, a published study characterizing proteins associated with activated CARD11 variants expressed in BJAB cells did not identify AP2 complex proteins [61]. To investigate whether AP2 was involved in CARD11 signalling in keratinocytes, a HaCaT-TR cell line was generated which expressed the activated CARD11 variant, CARD11^L232LI^-3xFLAG [14], after tetracycline induction.

**Figure 6.**
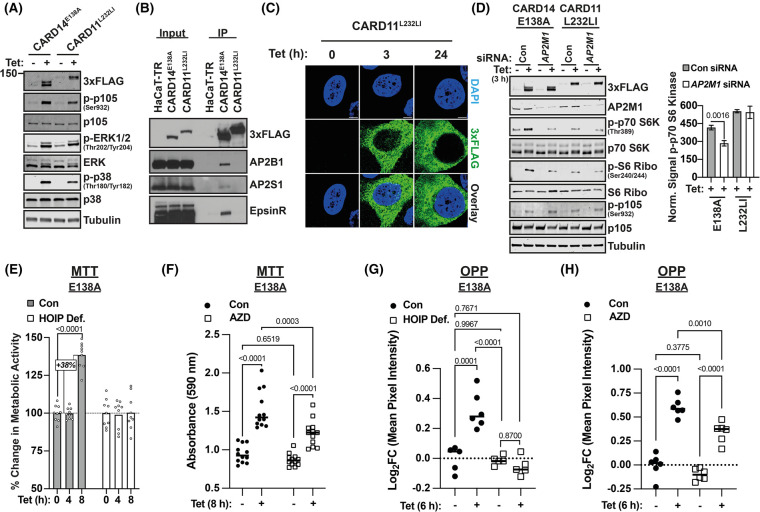
CARD11^L232LI^ activates mTORC1 independently of AP2M1 and CARD14^E138A^ induces cell metabolism via mTOR in HaCaT-TR cells. (**A**) Expression of CARD11^L232LI^-3xFLAG and CARD14^E138A^-3xFLAG were induced with tetracycline (Tet; 3 h) in HaCaT-TR cells. Cell lysates were immunoblotted for the indicated antigens. (**B**) Parental HaCaT-TR cells (HaCaT-TR), CARD14^E138A^-3xFLAG HaCaT-TR cells (CARD14^E138A^) and CARD11^L232LI^-3xFLAG HaCaT-TR cells (CARD11^L232LI^) were induced with tetracycline (Tet; 3 h). Cell lysates were immunoprecipitated with anti-FLAG. Immunoprecipitates (IP) and input lysates were immunoblotted for the indicated antigens. (**C**) Confocal imaging of anti-FLAG localization in CARD14^E138A^-3xFLAG HaCaT-TR cells cultured for the indicated times with tetracycline (Tet). Nuclei were stained with DAPI. (**D**) CARD14^E138A^-3xFLAG HaCaT-TR cells (CARD14^E138A^) and CARD11^L232LI^-3xFLAG HaCaT-TR cells (CARD11^L232LI^) were pre-treated with *AP2M1* or control (Con) siRNAs. Cell lysates were prepared after tetracycline induction (Tet; 3 h) and immunoblotted for the indicated antigens. S6 kinase phosphorylation was quantified and normalized to tubulin loading control for three independent experiments. Means (±SD) are presented and tested statistically by unpaired two-tailed *t*-test relative to control means for each cell line. (**E**) Triplicate cultures of HOIP-deficient (HOIP Def.) and control non-targeted (Con) CARD14^E138A^ 3xFLAG HaCaT-TR (Cas9) cells were induced for with tetracycline (Tet) for the indicated times. Metabolic activity was monitored by MTT assay (mean ± SD). (**F**) Triplicate cultures of CARD14^E138A^ 3xFLAG HaCaT-TR cells were induced with tetracycline (Tet; 8 h) plus AZD8055 (AZD) or DMSO vehicle control. Metabolic activity was monitored by MTT assay (mean ± SD). Statistical significance was tested by two tailed, unpaired *t*-test. (**G**) Triplicate cultures of HOIP-deficient (HOIP Def) and control non-targeted (Con) CARD14^E138A^ 3xFLAG HaCaT-TR (Cas9) cells were induced with tetracycline (Tet; 6 h). Nascent protein synthesis was monitored by OPP assay (mean ± SD). (**H**) Triplicate cultures of CARD14^E138A^ 3xFLAG HaCaT-TR cells were induced with tetracycline (Tet; 6 h) plus AZD8055 (AZD) or DMSO vehicle control. Nascent protein synthesis was monitored by OPP assay (mean ± SD). Statistical significance was tested by two tailed, unpaired *t*-test.

Similar to CARD14^E138A^-3xFLAG, CARD11^L232LI^-3xFLAG induced IKK phosphorylation of p105 and activation of p38α and ERK1/2 MAP kinases (Figure 6A). However, CARD11^L232LI^-3xFLAG did not co-immunoprecipitate with the AP2 complex components AP2B1 and AP2S1 or epsinR, in contrast with CARD14^E138A^-3xFLAG (Figure 6B). Furthermore, confocal imaging showed that CARD11^L232LI^-3xFLAG had a uniform granular distribution in the cytoplasm (Figure 6C), similar to CARD14^WT^-3xFLAG but distinct from the punctate distribution characteristic of CARD14^E138A^-3xFLAG (Figure 4B).

CARD11^L232LI^-3xFLAG expression in HaCaT-TR keratinocytes induced mTORC1 phosphorylation of S6 kinase and consequent phosphorylation of S6 like CARD14^E138A^-3xFLAG (Figure 6D). However, AP2M1 knockdown revealed that CARD11^L232LI^-3xFLAG induction of S6 kinase phosphorylation was independent of AP2 function, in contrast with CARD14^E138A^-3xFLAG (Figure 6D).

Together these results suggested that the targeting of activated CARD14 to early endosomes in keratinocytes and its functional association with AP2 for mTORC1 activation might be specific to this CARD-CC family member.

### CARD14^E138A^ induces mitochondrial metabolism in keratinocytes and epidermal acanthosis via mTOR

Psoriatic keratinocytes are characterized by their aberrant proliferation and differentiation [1]. mTOR co-ordinates eukaryotic cell growth and metabolism in response to the environment [55]. Thus, CARD14^E138A^ regulation of cell metabolism in HaCaT-TR keratinocytes was next determined. Tetracycline induction of CARD14^E138A^-3xFLAG increased mitochondrial metabolism (MTT assay monitoring cellular oxidoreductase enzymes) and nascent protein synthesis (OPP assay) in HaCaT-TR cells (Figure 6E–H). Total cell numbers and the fraction of Ki67^+^ cells did not change in these short-term 6–8 h assays (Supplementary Figure S6B,C). The effects of CARD14^E138A^-3xFLAG on mitochondrial metabolism and protein synthesis were completely blocked by the absence of HOIP (Figure 6E,G). The mTOR inhibitor AZD5363 also significantly reduced both of these increases showing that CARD14^E138A^ stimulated cell metabolism, in part, via its ability to activate mTOR kinase function (Figure 6F,H).

These results suggested that inhibition of mTORC1 might ameliorate the effects of CARD14^E138A^ on keratinocyte proliferation and differentiation in psoriasis. This was investigated by comparing Rosa26-LSL-*CARD14^E138A^*^/+^ K14-CreERT2 mouse strain (Tg CARD14^E138A^) that inducibly expresses CARD14^E138A^ in keratinocytes upon tamoxifen administration with control mice (Tg control) [19]. Analysis of S6 phosphorylation in skin sections from Tg CARD14^E138A^ mice demonstrated strong activation of mTOR signalling in acanthotic keratinocytes 5 days after initiating tamoxifen injection (Supplementary Figure S7). In contrast, skin sections from Tg control mice showed minimal phospho-S6 staining 5 days after tamoxifen injection (Supplementary Figure S7). Thus, CARD14^E138A^ activates mTORC1 in primary mouse keratinocytes, consistent with the HaCaT-TR keratinocyte experiments.

**Figure 7.**
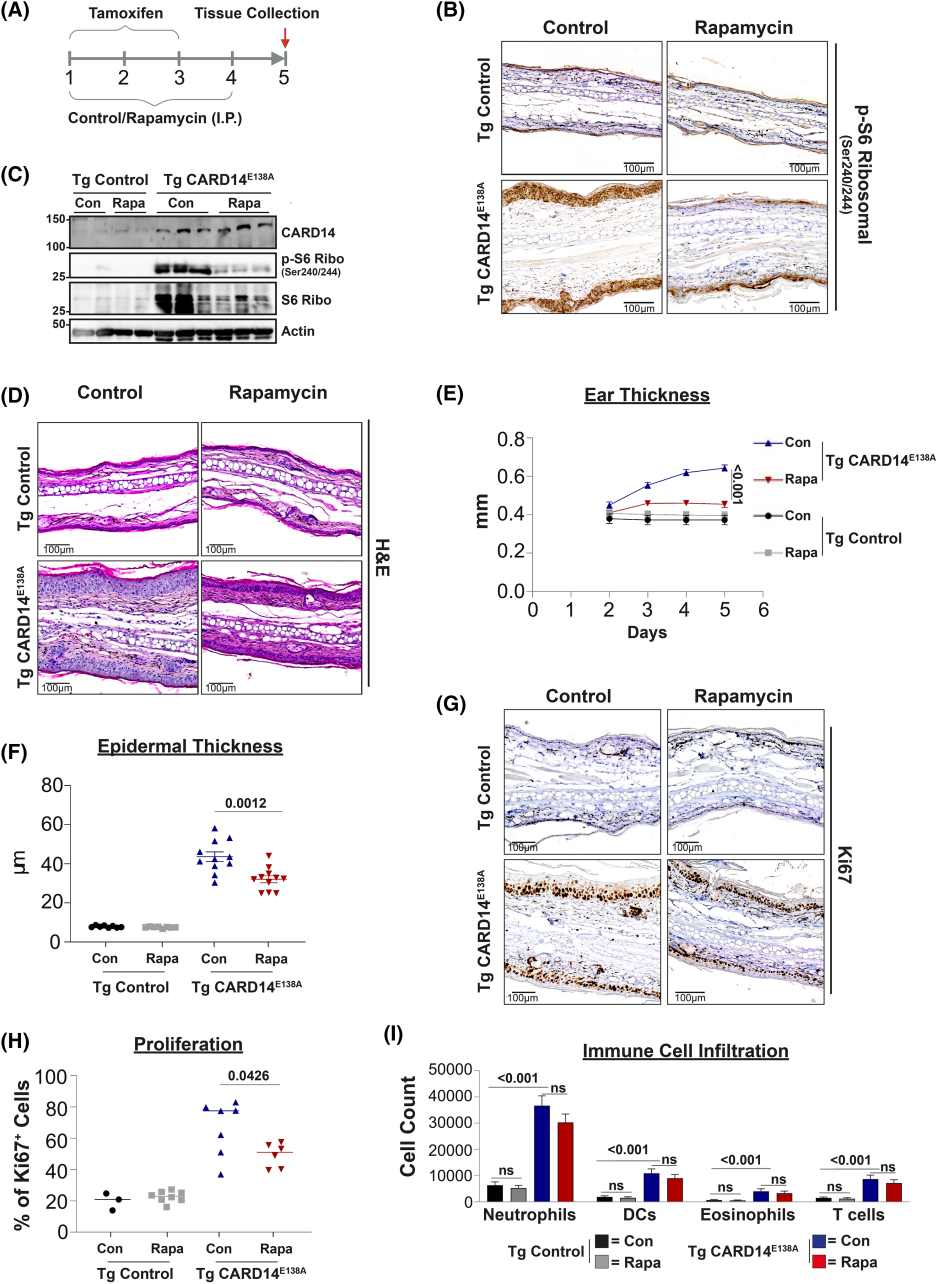
Rapamycin reduces CARD14^E138A^-induced epidermal acanthosis in mice. (**A**) Schematic representation of the experimental procedure for CARD14^E138A^ induction and rapamycin treatment. CARD14^E138A^ expression was induced in keratinocytes of Tg CARD14^E138A^ by daily tamoxifen injections up to 3 days. Rapamycin or control vehicle was injected daily intraperitoneally on d1–d4. Mice were culled on d5 for analysis. Tg control mice were injected with tamoxifen ± rapamycin for comparison. (**B**) Ear sections were produced from mice 5 days after tamoxifen induction ± vehicle (Control) or rapamycin co-injection. Representative sections are shown, stained with hematoxylin and eosin (H&E) and anti-phospho-S6. Scale bar represents 100 µM. (**C**) Skin extracts from ears were produced from mice 5d after tamoxifen induction ± vehicle control (Con) or rapamycin (Rapa) injection and immunoblotted for the indicated antigens. (**D**) Ear sections were produced from mice 5d after tamoxifen induction plus vehicle (Control) or rapamycin treatment. Representative images are shown of ear sections stained with H&E. Scale bar represents 100 µM. (**E**) Changes in ear thickness in mice treated with vehicle (Con) or rapamycin (Rapa) following tamoxifen induction of Tg CARD14^E138A^ and Tg Control mice. Combined results of two independent experiments are shown. Ear thickness data were analyzed as repeated measurements using the residual maximum likelihood (REML). (Tg Control/vehicle, *n* = 6; Tg Control/rapamycin *n* = 7, Tg CARD14^E138A^/vehicle, *n* = 10, Tg CARD14^E138A^/rapamycin *n* = 10). (**F**) Changes in epidermal thickness in Tg CARD14^E138A^ and Tg Control mice treated with vehicle (Con) or rapamycin (Rapa) following tamoxifen induction. Each symbol represents the mean of at least 10 epidermal thickness measurements for each ear, the line represents the mean value. (**E** and **F**) Statistical differences were determined using a modified *F*-test. *P* values shown. (**G**) Ear sections were produced from Tg CARD14^E138A^ and Tg Control mice 5d after tamoxifen induction plus vehicle (Control) or rapamycin treatment. Representative images are shown of ear sections stained with H&E and anti-Ki67. Scale bar represents 100 µM. (**H**) Quantification of Ki67 positive cells from control (Tg Control) and Tg CARD14^E138A^ mice treated with vehicle (Con) or rapamycin (Rapa). Each symbol represents one mouse, the line represents the mean value. Statistical difference between two groups was determined using one-way ANOVA test. *P < 0.01. (**I**) Analysis of infiltrating immune cells in single cell suspension of the ears from Tg Control and Tg CARD14^E138A^ mice treated or non-treated with rapamycin were analysed by flow cytometry. Cell counts of neutrophils (CD45^+^CD3^−^CD19^−^CD11b^+^Ly6G^+^), DCs (CD45^+^CD3/CD19^−^CD11b^+^CD64^−^MHCII^+^CD11c^+^), eosinophils (CD45^+^CD3/CD19^−^CD11b^+^CD64^−^SiglecF^+^), T-cells (CD45^+^CD3/CD19^+^,MHCII^−^). The data was log-transformed and analyzed using a modified *F*-test. Significant *P*-values shown. NS, not significant. (Tg Control/vehicle, *n* = 4; Tg Control/rapamycin, *n* = 4; Tg CARD14^E138A^/vehicle, *n* = 6; Tg CARD14^E138A^/rapamycin, *n* = 6).

To determine the importance of CARD14^E138A^ activation of mTORC1 in psoriasiform dermatitis, Tg CARD14^E138A^ and Tg control mice were injected intraperitoneally with rapamycin (Figure 7A). Analysis of S6 phosphorylation by immunocytochemistry and immunoblotting of skin extracts confirmed that rapamycin blocked tamoxifen-induced mTORC1 activation by CARD14^E138A^ in the skin (Figure 7B,C). Rapamycin treatment also significantly reduced ear and epidermal layer thickening induced by CARD14^E138A^, in comparison with vehicle control (Figure 7D–F). CARD14^E138A^ activation of mTORC1 in mouse skin keratinocytes was therefore required for epidermal acanthosis. In line with our earlier findings, acanthotic keratinocytes in tamoxifen-induced Tg CARD14^E138A^ mice stained for the proliferation marker Ki67 [23]. Rapamycin significantly reduced the percentage of Ki67^+^ staining keratinocytes (Figure 7G,H), demonstrating that CARD14^E138A^ activation of mTORC1 promoted keratinocyte proliferation. In contrast, flow cytometric analyses revealed that mTORC1 inhibition by rapamycin did not alter CARD14^E138A^-induced infiltration of immune cells into the skin (Figure 7I).

## Discussion

CARD14 signalling pathways have been poorly investigated and much of our knowledge is based on similarities with other CARD-CC family members [25]. Here, we have extensively characterized the acute effects of the psoriasis-associated, gain-of-function CARD14^E138A^ variant in HaCaT-TR keratinocytes. This revealed that the AP2 complex associated with the CARD14^E138A^ signalosome, which was found to be targeted to early endosomes. Knockdown experiments showed that AP2 function was not required for CARD14^E138A^ to activate NF-κB or MAPK, but was needed to activate mTORC1, a novel CARD14 signalling pathway identified in this study that mediated CARD14^E138A^-induced epidermal acanthosis. Similar mechanisms were involved in CARD14^E138A^ activation of NF-κB and MAPKs to those previously described for CARD11 [11,12]. However, an activated CARD11 mutant did not associate with AP2 or localize to endosomes and could activate mTORC1 independently of AP2 function in HaCaT keratinocytes. In addition, CARD14 activation of MALT1 protease activity was dependent on HOIP and TRAF6, in contrast with CARD11 [62]. Thus, CARD14 and CARD11 signalling mechanisms are not identical, perhaps reflecting the relatively low overall homology between these two proteins.

A striking feature of the CARD14^E138A^ signalosome was the number of negative regulators that were recruited and the transient nature of the downstream signalling induced. None of these regulators associated with CARD14^WT^ indicating CARD14 signalling was required for binding to CARD14^E138A^. Unregulated inflammation is harmful and it is likely that these negative regulators were part of a homeostatic response to turn off constitutive CARD14^E138A^ signalling. Consistent with this hypothesis, the recruitment of A20 and ABIN1 to the CARD14^E138A^ signalosome was dependent on the positive regulators HOIP and TRAF6. It is possible that the genetic association with genes encoding A20 and ABIN1 with psoriasis [7,34] is due, at least in part, to their direct inhibitory effects on CARD14 signalling. ABIN1 was identified as an A20 binding protein [63] and was suggested to inhibit TNFR1 signalling by recruiting A20 to the activated receptor complex [64]. In contrast, ABIN1 and A20 associated with CARD14^E138A^ and inhibited signalling independently of one another. Both A20 and ABIN1 regulated signalling by indirectly modulating CARD14^E138A^ levels. It will be important to confirm the importance of these and other negative regulators in CARD14 signalling in a more physiological system which does not involve CARD14^E138A^ overexpression.

TRAF2, functioning redundantly with TRAF6, promotes TCR-induced NF-κB activation [65]. In contrast, TRAF2 reduced CARD14^E138A^ activation of NF-κB in keratinocytes, stimulating CARD14^E138A^ turnover. TRAF2 appeared to function as an adaptor, which recruited cIAP1 to the CARD14^E138A^ signalosome to reduce CARD14^E138A^ expression. The RING domain of TRAF2 is not able to bind to E2 [66] and TRAF2 has been shown to have a similar adaptor function in the alternative pathway of NF-κB activation in B cells, associating with cIAPs to induce K48-linked ubiquitination and degradation of NIK, blocking NF-κB2 p100 processing [67]. cIAP1/2 function in NF-κB activation also differs between CARD11 and CARD14. In ABC-DLBCL addicted to chronic BCR signalling, cIAP1/2-induced K63-linked ubiquitination of BCL10 is required to recruit HOIP to the CARD11-BCL10-MALT1 complex to activate NF-κB [68]. In contrast, cIAP1 was not required for HOIP interaction with the CARD14^E138A^ signalosome. However, in the ABC DLBCL line OCI0-Ly3, cIAP1/2 is not required for NF-κB activation or survival triggered by mutant CARD11 [68].

Imaging experiments and PLAs indicated that activated CARD14^E138A^ was associated with early endosomes and moved along microtubules in the cytoplasm. Biochemical experiments also revealed a link between CARD14^E138A^ and intracellular trafficking, showing that CARD14^E138A^ associated with the AP2 complex, which is involved with clathrin-mediated endocytosis [32] and lysosome homeostasis [60]. Biochemical characterization of the CARD11^L232LI^ signalosome in BJAB cells did not reveal an association of activated CARD11 with AP2 [61]. However, CARD11 signalling in a subset of B cell lymphomas is localized to endocytic vesicles. In these cells, B cell receptor (BCR) signalling involves the formation of a multiprotein super-complex by the adaptor MyD88, TLR 9 and the BCR (termed the My-T-BCR supercomplex) [69]. My-T-BCR co-localises with mTOR on endolysosomes, where it promotes cell survival by activating NF-κB (via a CARD11-BCL10-MALT1 complex) and mTOR signalling. Inhibitors of mTOR signalling decrease the formation of the My-T-BCR super-complex, impairing activation of both NF-κB and mTOR. Interestingly, pulldown experiments indicate that My-T-BCR is also associated with the AP2 complex. This suggests that mTORC1 activation by CARD11 in the My-T-BCR supercomplex in B lymphoma cells may require AP2, similar to CARD14^E138A^ in keratinocytes. However, CARD14^E138A^ activation of mTOR was not dependent on NF-κB activation. CARD11 activation of mTOR following TCR stimulation is also independent of NF-κB activation [70,71].

We had previously reported that association of familial psoriasis with SNPs residing in an intron of *RPTOR* (encoding the RAPTOR component of mTORC1) is associated with the development of psoriasis [72,73], although this genetic association did not achieve genome-wide significance and is not seen in psoriasis case/control studies [74]. Nevertheless, mTOR signalling is hyper-activated in psoriatic lesions [75] and altered expression of RPTOR may contribute to psoriasis susceptibility. Furthermore, Akt, a key upstream regulator of mTORC1, is highly activated in suprabasal epidermal layers of psoriatic lesions [76]. *In vitro* experiments suggest that mTORC1 signalling be must be switched off for normal keratinocyte terminal differentiation, while the inflammatory environment of psoriasis activates mTORC1 resulting in aberrant keratinocyte differentiation [77]. We have demonstrated that CARD14^E138A^ activation of mTORC1 increased the mitochondrial metabolism and protein synthesis of HaCaT keratinocytes. CARD14^E138A^ activation of metabolism via mTORC1 will support the proliferation of keratinocytes, a hallmark of psoriasis lesions [78,79]. Psoratic keratinocytes are also characterized by resistance to apoptosis and by a senescent phenotype [80]. CARD14 activation of Akt will additionally maintain the survival of keratinocytes while promoting senescence [81]. The physiological significance of these findings is consistent with our demonstration that CARD14^E138A^-induced acanthosis and keratinocyte proliferation in mice was significantly reduced by rapamycin. Interestingly, rapamycin did not impair CARD14^E138A^-induced recruitment of immune cells into the skin. In line with this, induction of proinflammatory gene expression by CARD14^E138A^ in HaCaT-TR cells was not altered by inhibition of mTOR. Thus, CARD14^E138A^ promotes inflammation and acanthosis independently of one another, presumably via the independent downstream activation of IKK and mTORC1, respectively.

The strong genetic evidence linking CARD14 to psoriasis (https://platform.opentargets.org/disease/EFO_0000676/associations), combined with data from loss-of-function and gain-of-function *Card14* mutants in mice suggests that pharmacologic inhibition of CARD14 signalling will ameliorate psoriasis [16]. HOIP is essential for CARD14^E138A^ activation of downstream signalling pathways, indicating that LUBAC has positive roles in TNF-dependent psoriasisform dermatitis induced in mice by CARD14^E138A^ [23]. However, LUBAC also has a key role in preventing the development of dermatitis by inhibiting keratinocyte death induced by TNF, TRAIL and CD95L [82]. Similarly, IKK signalling in keratinocytes blocks TNF-induced apoptosis, preventing dermatitis [83]. These findings have shown that keratinocyte cell death is a potent trigger of skin inflammation and highlight the pro-inflammatory risk of using inhibitors that target cell survival signalling pathways (e.g. LUBAC or IKK2 inhibitors) for treatment of CARD14-dependent psoriasis. Based on *in vitro* experiments, we have previously suggested that MALT1 inhibitors might be a good alternative to block CARD14 signalling in psoriasis [17,18]. This was further supported by our demonstration that oral treatment of mice with a specific MALT1 protease inhibitor strongly reduces psoriatic skin disease in CARD14^E138A^ transgenic mice [19]. While long-term genetic MALT1 inhibition in mice is not deleterious [19], the selective MALT1 inhibitor MLT-943 has recently been shown to severely reduce regulatory T cell numbers, promoting an IPEX-like autoimmune pathology [84]. Consequently, inhibiting MALT1 to block CARD14 signalling in psoriasis may also not be a viable option. The results of the present study indicate that mTORC1 will be activated in psoriatic keratinocytes in which CARD14 signalling is switched on by mutation or upstream cytokine stimulation. Indeed, this may explain why mTORC1 is activated in lesional skin of psoriasis patients [75]. Rapamycin inhibition of mTORC1 signalling impairs the development of imiquimod-induced psoriasiform dermatitis in mice [85], which is partially dependent on CARD14 expression [86]. Furthermore, this study has demonstrated that inhibition of CARD14-mediated mTORC1 signalling in the skin with rapamycin ameliorated epidermal acanthosis in mice conditionally expressing CARD14^E138A^ in keratinocytes. Together these results suggest that small molecule inhibitors which block CARD14 activation of mTORC1 (e.g. PI 3-kinase, Akt or mTOR inhibitors) may have beneficial therapeutic effects in psoriasis in combination with an anti-inflammatory drug. Consistent with this idea, rapamycin (sirolimus) in combination with cyclosporine reduces psoriasis area and severity in patients with severe chronic plaque psoriasis [87].

In conclusion, this study has identified key components of the psoriatic CARD14 signalosome using a model HaCaT keratinocyte system, in which CARD14^E138A^ expression was induced by tetracycline. This highlighted the importance of ubiquitination in positive and negative regulation of CARD14 signalling. mTORC1 activation was also discovered as a new signalling output downstream of CARD14, directly linking mTORC1 signalling in psoriasis [88] to a key protein involved in psoriasis pathogenesis. These results suggest that CARD14 signalling in keratinocytes has two key functions in the development of psoriatic disease. Firstly, CARD14 activation of NF-κB and MAP kinases promotes activation and infiltration of immune cells in the skin by inducing pro-inflammatory gene expression. Secondly, CARD14 activation of mTORC1 supports increased keratinocyte proliferation and alters keratinocyte differentiation. The CARD14-mTOR signalling axis may be a promising therapeutic target for the treatment of psoriasis.

### Limitations of study

Investigating CARD14 signalling mechanisms has been hampered by the absence of good CARD14 antibodies that can detect the endogenous protein and by limited availability of patient keratinocytes harbouring CARD14 variants. To circumvent these limitations in this study, we developed an HaCaT keratinocyte model system in which tetracycline induces the expression of transfected CARD14-3xFLAG which has allowed the specific effects of the E138A alteration on inducing CARD14 interactions and signalling to be investigated. Importantly, expression of WT CARD14-3xFLAG does not trigger the formation of a signalosome or downstream signalling in this system. Nevertheless, it is possible that CARD14^E138A^-3xFLAG induction of multiple negative regulators was a response to strong signalling induced by overexpression. With the development of improved reagents, it will be important in future studies to confirm the roles of ubiquitination in CARD14 signalling triggered by activating mutations or receptor stimulation by analysis of endogenous CARD14 in primary keratinocytes.

## Materials and methods

### Antibodies and general reagents

The following antibodies were used: anti-FLAG (M2) (Sigma, #F1804, or #F3165); anti-tubulin (TAT-1); p-IKK1/2 (2078S); p-p105 (CST, #4806); RelA (CST, #8242); p-ERK1/2 (CST, #9101); p-p38 (CST, #4511); HOIP (Merck, #SAB2102031); HOIL-1/RBCK1 (Atlas Antibodies, #HPA024185); SHARPIN (Proteintech, #14626); TRAF2 (Abcam, #ab126758); TRAF6 (Abcam, #ab40675); BCL10 (SCBT, #sc-5273); MALT1 (SCBT, #sc-28246; CST, #2494); ABIN1 (Ubiquigent, #68-0002-100); A20 (CST, #5630S); DIABLO (CST, #D5S3R); cIAP1 (CST, #7065); cIAP2 (CST, #3130); TAK1 (CST, #4505S); IKK2 (CST, #2684); EpsinR (Thermo Fisher Scientific, #PA5-60308); AP2B1 (SCBT, #sc-58226); AP2A1 (Abcam, #ab128950); total ubiquitin (CST, #3933); M1-linked ubiquitin (Merck, LUB9, #MABS451); K48-linked ubiquitin (CST, #8081); p-TAK1 (CST, #4536); p-TAB2 (CST, #8155); p-TRAF2 (CST, #13908); p-A20 (CST, #63523); p-MKK4 (CST, #9156); p-MK2 (CST, #3007); p-MEK1/2 (CST, #9121); p-JNK (CST, #9255/9251); p-JUN (CST, #9261/9164); p-FRA1 (CST, #5841); p-ATF2 (CST, #9225); p-mTOR (CST, #2481/2488); p-p70 S6K (CST, #9234); p-eIF4B (CST, #3591); p-S6 Ribosomal (CST, #2215). Antibodies were used at dilutions indicated by manufacturers.

Chemicals and general reagents were purchased from Merck, unless stated otherwise.

### Generation of HaCaT cell lines

Tetracycline-inducible CARD14^WT^-3xFLAG and CARD14^E138A^-3xFLAG HaCaT-TR cells [89] were generated by transfection of the full-length human cDNAs, with C-terminal 3xFLAG or EGFP tag sequences, subcloned into the pcDNA5/TO vector (ThermoFisher Scientific, #V103320). This allowed recombinant CARD14 protein to be induced by culture in tetracycline-containing medium, as reported previously [18]. Tetracycline (Merck, #T7660) was added in serum-free media to a final concentration of 1 μg/ml.

To facilitate the generation of knockout cell lines, CARD14^E138A^ 3xFLAG HaCaT-TR cells were stably transfected with px459-Cas9-Puro (Addgene, #62998) and selected in puromycin (0.5 μg/ml). Clones were selected for Cas9 expression by immunofluorescence staining (Abcam, #ab204448). Positive clones were then checked for CARD14^E138A^-induced RelA translocation (see below) following tetracycline addition, similar to the parental CARD14^E138A^-3xFLAG HaCaT-TR cells. Validated clones were used to generate knockout sublines lacking expression of specific CARD14^E138A^ interacting proteins, via crRNA and tracrRNA transfection. Four individual Edit-R crRNAs per target gene were purchased from Dharmacon. Specifically, Edit-R crRNAs: *TRAF6* (CM-004712-01/2/3/4), *RNF31* (CM-021419-01/2/3/4), *TNFAIP3* (CM-009919-01/2/3/4), *TNIP1* (CM-016358-01/2/3/4), *TRAF2* (CM-005198-01/2/3/4), *BIRC3* (CM-004099-01/2/3/4). Individual crRNAs were pooled and combined with Edit-R tracrRNA (GE, Dharmacon, #U-002005) in Opti-MEM. Pooled crRNAs and tracrRNA each had a final well concentration of 10 nM (2.5 nM per crRNA). Lullaby transfection reagent (OZ Biosciences, #LL71000) was used for reverse transfection. Monoclonal cell lines were isolated limiting dilution culture. Successful gene disruption was confirmed by immunoblotting.

### siRNA knockdown

All siRNA knockdown experiments were carried out using reverse transfection of cells. The transfection reagent mixture consisted of ON-TARGETplus siRNA SMARTpools (Horizon Discovery/Dharmacon) mixed with Lullaby (OZ Biosciences, #LL71000) or INTERFERin-HTS (Polyplus, #410-015) transfection reagents in Optimem-MEM (ThermoFisher Scientific/Gibco, #31985062). This mixture was added to culture wells prior of cell seeding, according to manufacturer's instructions. Cells were then cultured for 72 h before tetracycline addition.

### Cell culture

HaCaT stable cell lines were maintained in ‘selection media’: DMEM (ThermoFisher Scientific, #41966) with 10% foetal calf serum (FCS) (Biosera, #FB-1001) and 10 000 units penicillin, 10 mg/ml streptomycin, and 200 mM l-glutamine (Merck, #G6784) with 10 μg/ml Blasticidin (for pcDNA6/TR; Invitrogen, #ant-bl-05) and Hygromycin B (for pcDNA5/TO containing inserted sequences, Invitrogen, #ant-hg-05). Cells were detached with Accutase according to manufacturer's instructions (Merck, #A6964) for experimental use and passaging. Experiments were performed after a minimum of two passages after thawing from liquid nitrogen.

### Inhibitors

The signalling inhibitors used and working concentrations are detailed in study legends and the key resources table. DMSO was used as a vehicle control. MG-132 was used to block proteasome function, while Bafilomycin A1 was used to inhibit lysosome-mediated proteolysis. The Smac-mimetic birinapant (TL32711) was used to deplete cIAP1.

### RNA sequencing

CARD14^E138A^-3xFLAG HaCaT-TR, CARD14^WT^-3XFLAG and HaCaT-TR (parental) keratinocytes were seeded (in triplicate) for tetracycline incubations of 0, 3, 6, and 9 h (as detailed in Supplementary Figure S1). Cells were lysed and RNA isolated (RNeasy kit, QIAGEN, #74106). mRNA was then purified using a poly-A KAPA mRNA Hyper Prep kit (Roche) and sequenced on an Illumina HiSeq4000 instrument. Sequencing adaptors were removed with ‘Trim Galore!’ utility version (0.4.2), which was also used to trim individual reads (q-parameter set to 20). Sequencing reads were aligned to the human genome and transcriptome (Ensembl) using RSEM version (1.3.0) with STAR aligner version (2.5.2) [90,91]. Sequencing quality of individual samples was assessed with FASTQC (version 0.11.5) and RNA-SeQC (version 1.1.8) [92]. R-Bioconductor package DESeq2 (version 1.14.1) was used to determine differential gene expression [93]. Gene set enrichment analysis was performed as described [94].

### Protein analysis

After experimental treatments, cells were washed in ice cold phosphate buffered saline (PBS) and lysed in RIPA buffer (50 mM Tris HCL pH 7.5, 150 mM NaCl, 2 mM EDTA, 2 mM sodium pyrophosphate, 50 mM sodium fluoride, 0.1% SDS, 0.5% Triton X-100, 0.5% deoxycholate, and protease inhibitor cocktail (Merck/Roche, #11836170001)). Lysates were then subjected to a freeze thaw cycle and cleared by centrifugation (18 000 ***g***) for 15 min at 4°C. The cleared soluble fraction was aspirated and mixed with 4× concentrated Laemmli sample buffer. For solubility comparisons, insoluble pellet extracted by boiling in 1X SDS-sample buffer. To monitor nuclear translocation of RelA, cells were extracted with NE-PER nuclear and cytoplasmic extraction buffers (ThermoFisher Scientific, #78833) used according to manufacturer's instructions.

SDS–PAGE was carried out using a Bio-Rad gel electrophoresis system and proteins transferred to PVDF membranes using a Bio-Rad Trans-Blot Turbo semi-dry transfer system (Bio-Rad, #1704157). Bound protein was detected by immunoblotting and enhanced chemiluminescence (ECL), using SuperSignal (ThermoFisher Scientific, #34095), Immobilon (Millipore, #WBKLS0500) or Amersham (GE Healthcare, #RPN2232) ECL reagents, as appropriate. For protein quantitation of western blots, the Odyssey M imaging system (LI-COR Inc., Lincoln, NE, U.S.A.) was used in conjunction with the fluorescently labelled secondary antibodies (LI-COR, #926-32212, #926-68073). Analysis of signals from these measurements was carried out using LI-COR Image Studio™ Software according to manufacturer's protocol.

### Immunoprecipitation of complexes

Forty-eight hours after seeding in 90 mm dishes, cells were induced with tetracycline for 3 h. After washing with PBS, cells were lysed in co-immunoprecpitation lysis buffer (150 mM NaCl, 50 mM Tris pH 7.5, 10% glycerol, 0.25% Triton X-100, 50 mM sodium fluoride, 5 mM sodium pyrophosphate, 10 mM beta-glycerolphosphate, 2 mM EDTA, 10 mM iodoacetamide, 0.1 mM sodium vanadate plus protease inhibitors). Lysates were cleared by centrifugation, protein amounts determined by Bradford assay (ThermoFisher Scientific, #23236) and normalized. After pre-clearing with mouse IgG-Agarose (Merck, #A0919), lysates were subjected to immunoprecipitation with anti-FLAG M2 antibody affinity agarose gel (Sigma–Aldrich #A2220) at 4°C for 2 h. After washing gel in lysis buffer, immunoprecipitated protein was eluted from M2 antibody resin with low pH buffer (0.2 M glycine, 0.05% NP-40, pH 2.5). Eluted protein was resolved by SDS–PAGE and immunoblotted.

### Mass spectrometry

Cells were cultured for at least seven doublings in Stable Isotope Labelling by Amino acids in Cell culture (SILAC) media (DMEM [Thermo Fisher Scientific, #A33832], 10% FCS dialyzed 2000 molecular mass cut-off) containing either heavy or light arginine and lysine (R0/R10, K0/K8; 0.1 mg/ml).

To analyze the CARD14^WT^-3xFLAG and CARD14^E138A^-3x-FLAG interactomes, HaCaT-TR control cells or CARD14^E138A/WT^ 3xFLAG HaCaT-TR cells were grown in heavy or light amino acid SILAC media. Three hours after tetracycline addition, washed cells were flash frozen in liquid nitrogen and stored at −80°C. Cells were thawed and lysed in detergent-free urea lysis buffer (8 M urea, 50 mM HEPES, 10 mM glycerol 2-phosphate, 50 mM sodium fluoride, 5 mM sodium pyrophosphate, 1 mM EDTA, 1 mM sodium vanadate, 1 mM dithiothreitol (DTT), 1 mM phenylmethanesulfonyl fluoride, 1 μg/ml aprotinin, 1 μg/ml leupeptin, 100 nM okadaic acid). Lysates were subjected to immunoprecipitation with anti-FLAG. Eluates from individual immunoprecipitations were mixed to create separate 1:1 mixes according to the Supplementary Table (Supplementary Figure S2A). Isolated protein was resolved by SDS–PAGE (12% NuPAGE, Invitrogen). Gel lanes were cut into pieces and subjected to in-gel digestion with trypsin (Pierce/Promega). Peptides were extracted in 0.1% formic acid, separated on a 50 cm × 75 μm I.D. EasySpray C18 column (ThermoFisher Scientific) and eluted directly onto an Orbitrap Fusion Lumos mass spectrometer coupled to an Ultimate 3000 uHPLC (ThermoFisher Scientific). MS data were searched against a UniProt *Homo sapiens* database with the Andromeda peptide search engine. MaxQuant v1.6.0.1 was utilized for data processing, using SILAC quantification algorithm [95,96], and imported into Perseus software (v1.4.0.2) for further statistical processing [97]. Estimated false discovery rates (FDR) for protein and peptides were set to 1%. Perseus software was used for further analyses and data visualization, with correlation plots generated to show log_2_ ratios of two separate SILAC comparisons [95].

For characterization of the CARD14^E138A^ phosphoproteome, cells were washed and lysed in detergent-free urea lysis buffer (see above). Four mixes in total were analyzed: two control and two experimental, comparing the parental cell line HaCaT-TR cultured in heavy or light amino acid SILAC media with CARD14^E138A^ 3xFLAG HaCaT-TR keratinocytes cultured in heavy or light media. As determined with the Bradford assay, mixes were normalized as: 2.5 mg each of heavy and light condition, for a total of 5 mg in each mix (Supplementary Figure S2A). Heavy-light mixes were generated by mixing normalized samples (1:1). Five milligrams of mixtures were digested with a combination of trypsin and LysC (Promega). After chromatography on a C18 Sep-Pak column (Waters), phosphopeptides were enriched using a High-Select Fe-NTA phosphopeptide enrichment kit (ThermoFisher Scientific, #A32992), according to manufacturer's instructions. Enriched phosphopeptides (in 1% trifluoroacetic acid) were separated on an ID PepMap column (C18, high pH fractionation; ThermoFisher Scientific) and eluted directly into an Orbitrap Fusion Lumos mass spectrometer (ThermoFisher Scientific). Data was acquired with Xcalibur software (ThermoFisher Scientific) and a PhosphoSTYsites.txt file imported into Perseus for downstream analyses.

### Microscopy

Keratinocytes were seeded on coverslips and recombinant CARD14 protein was induced with tetracycline (3 h) after 48 h culture. Cells were then fixed with 4% formaldehyde (10 min at room temperature (RT)) and permeabilized with 0.1% Triton X in PBS (4 min at RT). Antibody staining was carried out after blocking with fish skin gelatin (Merck, #G7041; 0.2% v/v in PBS). Secondary antibodies were diluted in blocking buffer containing DAPI counter stain. Images were acquired on a Zeiss LSM 780 or 880 confocal microscopes.

### Imaging of RelA nuclear translocation

Cells were seeded in optical culture plates (Greiner 384 well Flat Bottom TC-treated Imaging Microplate, #781091). Tetracycline induction of CARD14^E138A^ 3xFLAG expression was carried out for the indicated times following overnight culture. In experiments involving siRNA knockdown, cells were seeded onto transfection mixtures and tetracycline induction carried out after 72 h culture. Cells were fixed in 4% formaldehyde in PBS, washed with PBS and then permeabilized with 0.5% Triton X-100 in PBS. Following PBS washing, fixed cells were incubated with 3% bovine serum albumin in PBS to block non-specific protein binding. Cells were incubated in primary RelA antibody solution (1:400; Cell Signalling Tech, #8242) for 1 h at RT. After PBS washing, cells were incubated for 1 h with secondary antibody mixture (AlexaFluor-488/647-conjugated anti-rabbit Ig [Thermo Fisher Scientific, #A-11008] diluted 1/1000 in PBS-1% bovine serum albumin plus 1/1000 DAPI [Thermo Fisher Scientific, #D1306] to stain nuclei). After PBS washing, plates were sealed for analysis by fluorescence imaging (Cell Insight NXT High-Content Microscope [Thermo Fisher Scientific], LED illumination, ×10 magnification, and a 14 bit; 100 CCD camera). An ArrayScan VTI HCS and Cell Insight CX7 HCS reader was used for some experiments at equivalent settings. Thermo Scientific Compartmental Analysis V.4 BioApplication was used to define and quantify subcellular localization of RelA. Image analysis in conjunction with DAPI staining was utilized to define a masked region of the nucleus. The software was then used to define the cytoplasmic region with a width of 20 pixels from the edge of the nuclear mask. The BioApplication software was then used to measure the pixel intensities within these defined regions and generate well-averaged values of all cells within each well. Average values produced from each well represents at least nine images acquired per well in a grid. The measured difference in average intensity of nuclear RelA versus cytoplasmic RelA was plotted.

### Live cell imaging

CARD14^E138A^-EGFP HaCaT-TR cells were used for live cell imaging experiments. These were seeded in 35 mm (#1.5 thickness) glass bottomed dishes (MaTtek corporation, # P35G-1.5-14-C) with Fluorobrite (Gibco, Thermo Fisher Scientific, #A1896701) media. After 48 h, cells were cultured for 3 h with tetracycline to induce CARD14^E138A^-EGFP expression. SiR-tubulin was added (Cytoskeleton, Inc., #CY-SC002; 0.125 μM) for the last 5 h of culture to stain microtubules. Cells were imaged using a Nikon CSU-W1 spinning confocal microscope (Nikon Instruments Inc.) with an environmentally controlled incubation chamber. ImageJ (Fiji) with the TrackMate plugin was used to display and analyse acquisitions, with 90 ms exposures, ∼10 frames per second [98].

### Quantitative PCR

Keratinocytes were cultured with tetracycline for 6 h to induce gene expression, unless otherwise stated. Real-time quantitative reverse transcription PCR was performed using the TaqMan assay system (ThermoFisher Scientific/Applied Biosystems). TaqMan FAM-MGB probes were purchased from ThermoFisher Scientific: 18S (Hs99999901_s1), CCL20 (Hs01011368_m1), IL36G (Hs00219742_m1), CXCL1 (Hs00236937_m1), CXCL8 (Hs00174103_m1). Custom FAM-MGB primers were generated to specifically monitor the levels of expression of the sequence for stably-integrated CARD14^E138A^-3xFLAG using the TaqMan® Custom Plus Gene Expression Assay. The primers for this covered both the extreme C-terminal sequence of CARD14^E138A^ and the 3xFLAG tag sequence. All expression levels were normalized to the *18S* housekeeping gene, using the 2(−ΔΔCT) method.

### Metabolism and cell proliferation assays

For measurement of cell metabolic activity, a 3-(4,5-dimethylthiazol-2-yl)-2,5-diphenyltetrazolium bromide (MTT) assay was used (ab211091, Abcam), which measures the conversion of MTT to formazan by cellular NADH-dependent oxidoreductase enzymes. Replicate assays were carried out according to manufacturer's instructions, monitoring absorbance at 590 nm.

For measurement of nascent protein synthesis, an O-propargyl-puromycin (OPP) assay was used according to manufacturer's instructions (Click-iT™ Plus OPP Alexa Fluor™ 488 Protein Synthesis Assay Kit, C10456, ThermoFisher Scientific). In this assay, OPP was incorporated into nascent proteins, which were observed by fluorescence using a Zeiss LSM 780 microscope. DAPI nuclear staining was used to eliminate nuclear regions of the cell (ImageJ, FIJI software), allowing intensity measurements to be recorded from the cytoplasm. Each data point represents the average of one of several fields of view per slide/condition.

To monitor Ki67 expression, CARD14^E138A^-3xFLAG HaCaT-TR cells (triplicates) were cultured in optical quality 96-well plates (Nunc) ± tetracycline. Cells were stained with anti-Ki67 primary and IRDye 800CW anti-mIg secondary antibodies (LI-COR 926-32211) for an In-Cell Western Assay using a LI-COR Odyssey M imager. The Ki67 signal was normalized to cell numbers by CellTag 700 staining (LI-COR).

### Mouse experiments

Rosa26^LSL-CARD14-E138A^ K14creERT2 (CARD14^E138A^ Tg) mice have been described previously [19]. Eight to 12 week-old sex matched littermates were used in all experiments. Mice were maintained under specific pathogen-free conditions and were housed in individually ventilated cages under 12 h light/12 h dark cycles condition. Mice were given ad libitum access to sterile food and autoclaved water. All experiments were performed in accordance with institution, national and European animal guidelines. Animal protocols were approved by the ethics committee of Ghent University.

To induce expression of the *CARD14*^E138A^ transgene, both male and female mice of 8–12 weeks old were orally gavaged on three consecutive days with 2.5 mg tamoxifen (T-5648, Sigma–Aldrich) dissolved in corn oil (C8267, Sigma–Aldrich). To inhibit mTORC1, mice were treated daily for 4 days with rapamycin (tlrl-rap, Cayla Invivogen Europe), injected intraperitoneally at 6 mg/kg. Ear thickness was measured every day using a G-1A dial thickness gauge (Peacock, Japan) by an observer blinded to the treatments and genotypes. Mice were sacrificed on day 5 and ears were collected for further analysis.

### Histology

Ear tissue was fixed in 4% paraformaldehyde in PBS overnight at 4°C, followed by dehydration and embedding in paraffin. Sections of 5 μm were deparaffinized and then stained with hematoxylin and eosin using an Autostainer XL (Leica). For immunohistochemistry staining, sections were deparaffinized and hydrated. Antigen retrieval was performed by heating the slides in a citrate-based antigen unmasking solution (H-3300, Vector Laboratories) and endogenous peroxidase activity was blocked by immersing slides in 3% H_2_O_2_ in methanol. Nonspecific binding was prevented by incubating sections in 5% normal goat serum and 1% BSA. The following primary antibodies were used: anti-phospho S6 antibody (dilution 1:2000; D68F8 Rabbit mAB 5364S Cell Signalling) and anti-Ki67 antibody (dilution 1:1000; D3B5 Rabbit mAB 12202 Cell Signalling). After overnight incubation with primary antibody at 4°C, tissue sections were sequentially incubated with a biotinylated anti-rabbit secondary antibody (E0432, Dako), followed by incubation with Vectastain Elite ABC kit (PK-6100, Vector Laboratories), followed by detection with diaminobenzidine chromogen (ImmPACT DAB, SK-4105, Vector Laboratories) and counterstaining with hematoxylin. Sections were mounted by use of Entellan mounting medium (Merck Millipore). Images were acquired with Axio Scan.Z1 slide Scanner (Zeiss) and analyzed by using Zen Lite(Zeiss) software. Average epidermal thickness was determined as the mean of at least 10 measurements for each sample.

### Western blotting

After sacrifice, ears were collected, snap frozen and stored at −70°C. For analysis, tissue was homogenized using Precellys 24 (Bertin technologies with CK26 beads) in Laemmli buffer (50 mM Tris–HCl pH 8, 2% SDS, 10% glycerol). Debris was removed by two centrifugation steps at 16 000 ***g*** for 10 min. Protein concentration was determined using Pierce™ BCA Protein Assay Kit (23225, ThermoFisher Scientific) and equal amounts of protein extract were separated by 10% SDS–PAGE. Proteins were then transferred to nitrocellulose membranes (0.45 µm pore size; Protran, PerkinElmer). Membranes were blocked in 5% milk powder in TBS/0.2% Tween 20 (TBST) for 1 h at RT and probed with specific primary antibodies in 5% milk powder in TBST overnight at 4°C. The following antibodies were used: anti-CARD14 (10400-1-AP; Proteintech), Phospho-S6 Ribosomal Protein (D68F8 Rabbit mAB 5364S; Cell Signalling), S6 Ribosomal Protein (2317; Cell Signalling Technology), anti-actin monoclonal antibody (MP6472J; MP Biomedicals), secondary HRP-conjugated anti-mouse or anti-rabbit IgG antibody (31432 and 31464, respectively; ThermoFisher Scientific). Bound secondary antibodies were detected using the Western Lightning ECL detection system (PerkinElmer) according to the manufacturer's instructions.

### Statistical analysis

For experiments with Rosa26^LSL-CARD14-E138A^ K14creERT2 mice, ear thickness, epidermal thickness and cell counts were analysed using Genstat v22 (VSN International) and quantification of Ki67 positive cells was done using one-way ANOVA in GraphPad Prism version 9. Briefly, ear thickness, measured at consecutive equally spaced time points, was analyzed as repeated measurements using the residual maximum likelihood (REML) approach, as implemented in Genstat v22 (VSN International). A linear mixed model of the following form: response = μ + replicate + T1 + genotype_treatment + time + genotype_treatment.time + subject.time was fitted to the data. The term T1 is a covariate representing the ear thickness (mm) at the beginning of the experiment (before any treatment was applied). The fixed term genotype_treatment has four levels referring to the four possible combinations between genotype (WT and E138A(TG)) and treatment (control and rapamycin). The interaction term subject.time represents the residual error term with dependent errors because the repeated measurements are taken on the same subject, causing correlations among observations. Times of measurement were set at equal intervals. The autoregressive correlation structure (AR) was selected as best correlation model to account for the serial correlation between measurements within subjects. Significances of the fixed main effects, two-way interaction terms and pairwise comparisons between genotype treatment effects over time, were assessed by a modified *F*-test. A linear mixed model (random terms underlined) of the form: response = Constant + experiment + ear + genotype + treatment + genotype.treatment + genotype.treatment.ear + error, was fitted to the epidermal thickness data. Modified F statistics were used to assess the significance of the main and interaction effects. For the cell counts, a log-linear model of the form Constant + celltype × genotype × treatment was fitted using Genstat v22. The dispersion parameter for the variance of the response was estimated from the residual mean square of the fitted model. Wald statistics were used to assess the significance of the main and interaction effects, by dropping these fixed terms from the full model. Because all interaction terms were not significant, finally a reduced log-linear model of the form Constant + celltype + genotype + treatment was fitted to the cell counts.

## Data Availability

All of the raw RNA sequencing datasets of this study have been deposited in GEO: accession number GSE200493. The mass spectrometry data have been deposited to the ProteomeXchange Consortium via the PRIDE partner repository: interactome dataset identifier, PXD033039; phosphoproteomics dataset identifier, PXD033009.

## Open Access

Open access for this article was enabled by the participation of University College London in an all-inclusive *Read & Publish* agreement with Portland Press and the Biochemical Society under a transformative agreement with JISC.

## Abbeviations

ECL: enhanced chemiluminescence
FCS: foetal calf serum
FDR: false discovery rates
GWAS: genome-wide association studies
ID: inhibitory domain
MS: mass spectrometry
mTORC1: mTOR complex 1
OPP: *O*-propargyl-puromycin
PBS: phosphate buffered saline
PLA: proximity ligation assay
RT: room temperature
TLR: Toll-like receptor
WT: wild type
