# Supplementary material for: CARD14 signalosome formation is associated with its endosomal relocation and mTORC1-induced keratinocyte proliferation
Source: Biochem J. 2024 Sep 6;481(18):1143–71. doi: 10.1042/BCJ20240058 (PMC11555713; doi:10.1042/BCJ20240058)
Supplement: Supplementary Material 1 [file BCJ-481-1143-s1.pdf]

S1.

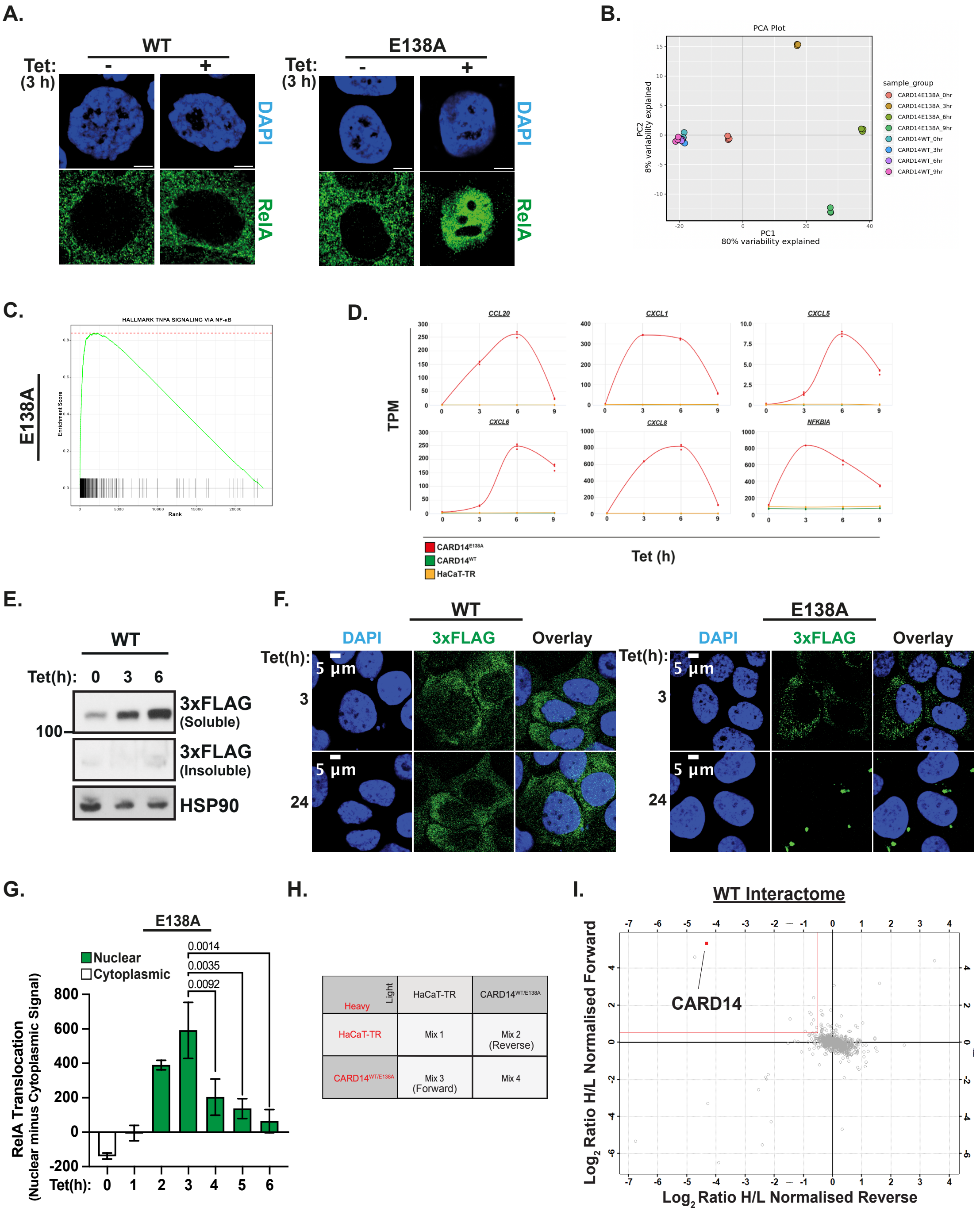

A.
WT
E138A
Tet:
(3 h)
-
+
DAPI
RelA
B.
PCA Plot
PC2
8% variability explained
PC1
80% variability explained
sample_group
CARD14E138A_0hr
CARD14E138A_3hr
CARD14E138A_6hr
CARD14E138A_9hr
CARD14WT_0hr
CARD14WT_3hr
CARD14WT_6hr
CARD14WT_9hr
C.
HALLMARK TNFA SIGNALING VIA NF-κB
E138A
Enrichment Score
Rank
D.
CCL20
CXCL1
CXCL5
CXCL6
CXCL8
NFKBIA
TPM
CARD14E138A
CARD14WT
HaCaT-TR
Tet (h)
E.
WT
Tet(h): 0 3 6
100
3xFLAG (Soluble)
3xFLAG (Insoluble)
HSP90
F.
WT
E138A
DAPI
3xFLAG
Overlay
Tet(h):
3
24
5 µm
G.
E138A
Nuclear
Cytoplasmic
0.0014
0.0035
0.0092
RelA Translocation
(Nuclear minus Cytoplasmic Signal)
Tet(h): 0 1 2 3 4 5 6
H.
Light
Heavy
HaCaT-TR
CARD14WT/E138A
Mix 1
Mix 2 (Reverse)
Mix 3 (Forward)
Mix 4
I.
WT Interactome
CARD14
Log2 Ratio H/L Normalised Forward
Log2 Ratio H/L Normalised Reverse

S2.

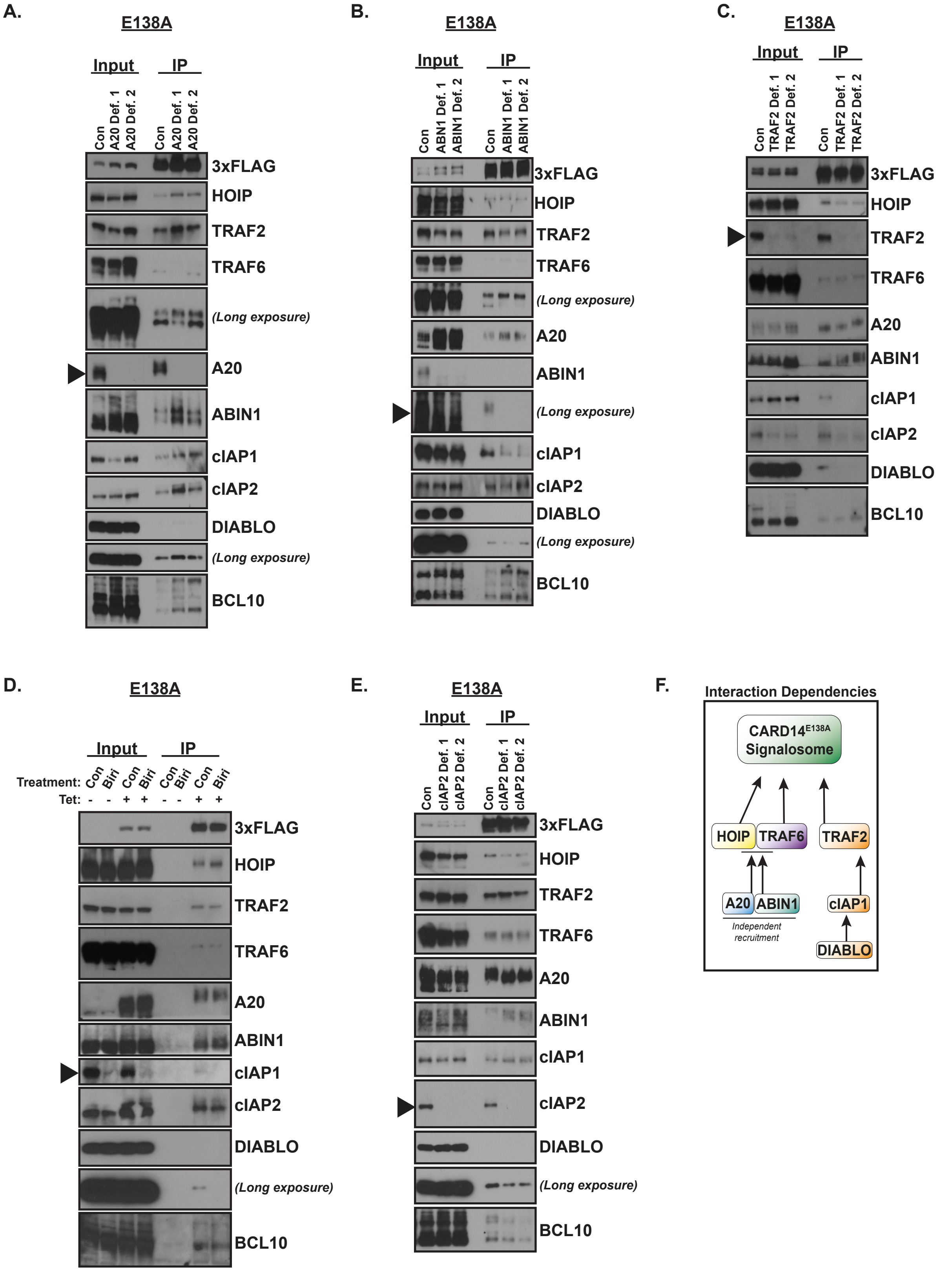
A.
E138A
Input
IP
Con
A20 Def. 1
A20 Def. 2
Con
A20 Def. 1
A20 Def. 2
3xFLAG
HOIP
TRAF2
TRAF6
(Long exposure)
A20
ABIN1
cIAP1
cIAP2
DIABLO
(Long exposure)
BCL10
B.
E138A
Input
IP
Con
ABIN1 Def. 1
ABIN1 Def. 2
Con
ABIN1 Def. 1
ABIN1 Def. 2
3xFLAG
HOIP
TRAF2
TRAF6
(Long exposure)
A20
ABIN1
(Long exposure)
cIAP1
cIAP2
DIABLO
(Long exposure)
BCL10
C.
E138A
Input
IP
Con
TRAF2 Def. 1
TRAF2 Def. 2
Con
TRAF2 Def. 1
TRAF2 Def. 2
3xFLAG
HOIP
TRAF2
TRAF6
A20
ABIN1
cIAP1
cIAP2
DIABLO
BCL10
D.
E138A
Input
IP
Treatment: Con Biri Con Biri Con Biri Con Biri
Tet: - - + + - - + +
3xFLAG
HOIP
TRAF2
TRAF6
A20
ABIN1
cIAP1
cIAP2
DIABLO
(Long exposure)
BCL10
E.
E138A
Input
IP
Con
cIAP2 Def. 1
cIAP2 Def. 2
Con
cIAP2 Def. 1
cIAP2 Def. 2
3xFLAG
HOIP
TRAF2
TRAF6
A20
ABIN1
cIAP1
cIAP2
DIABLO
(Long exposure)
BCL10
F.
Interaction Dependencies
CARD14E138A Signalosome
HOIP
TRAF6
TRAF2
A20
ABIN1
cIAP1
DIABLO
Independent recruitment

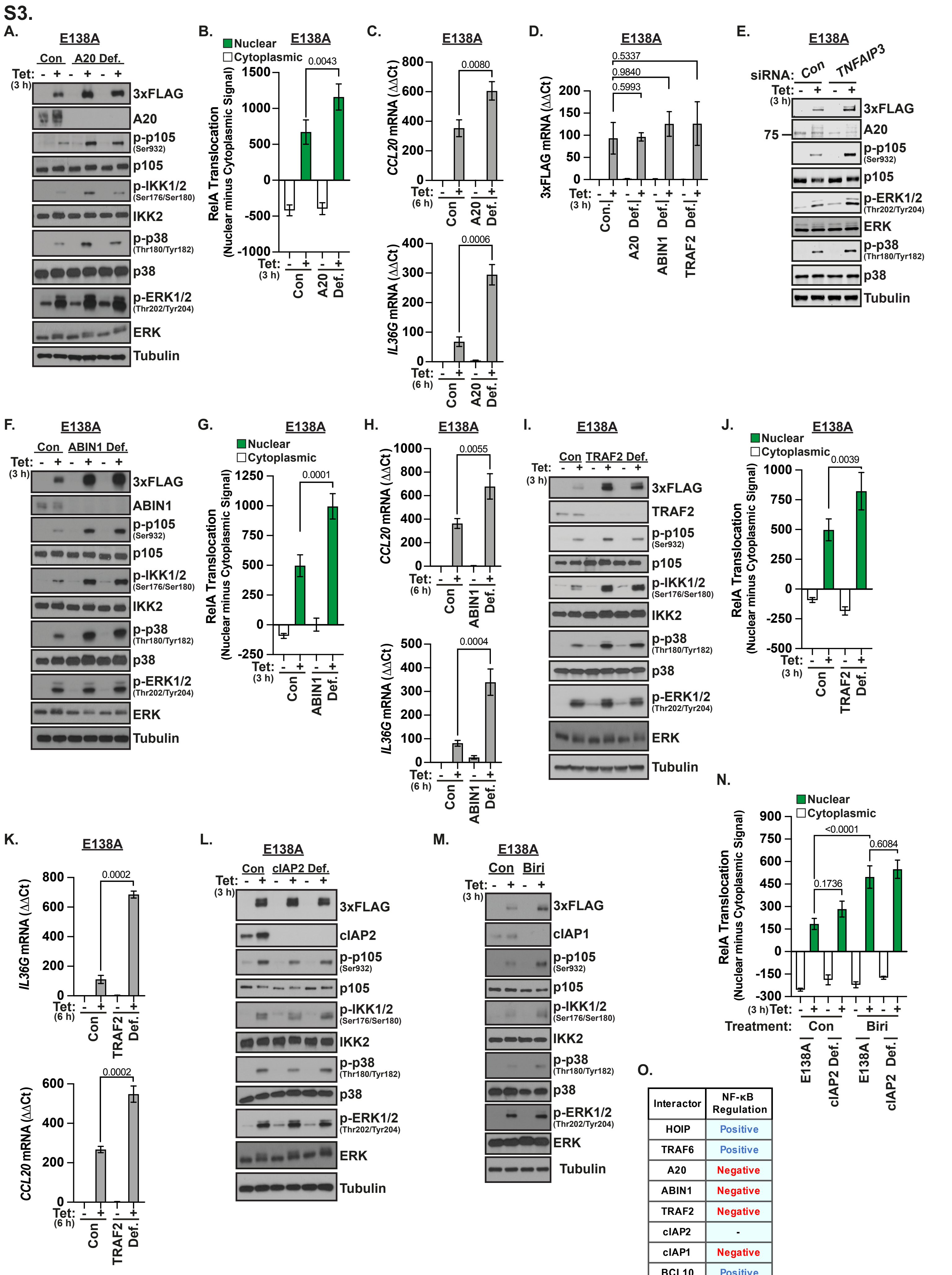

Interactor	NF-κB Regulation
HOIP	Positive
TRAF6	Positive
A20	Negative
ABIN1	Negative
TRAF2	Negative
cIAP2	-
cIAP1	Negative
BCL10	Positive
MALT1	Positive

S4.

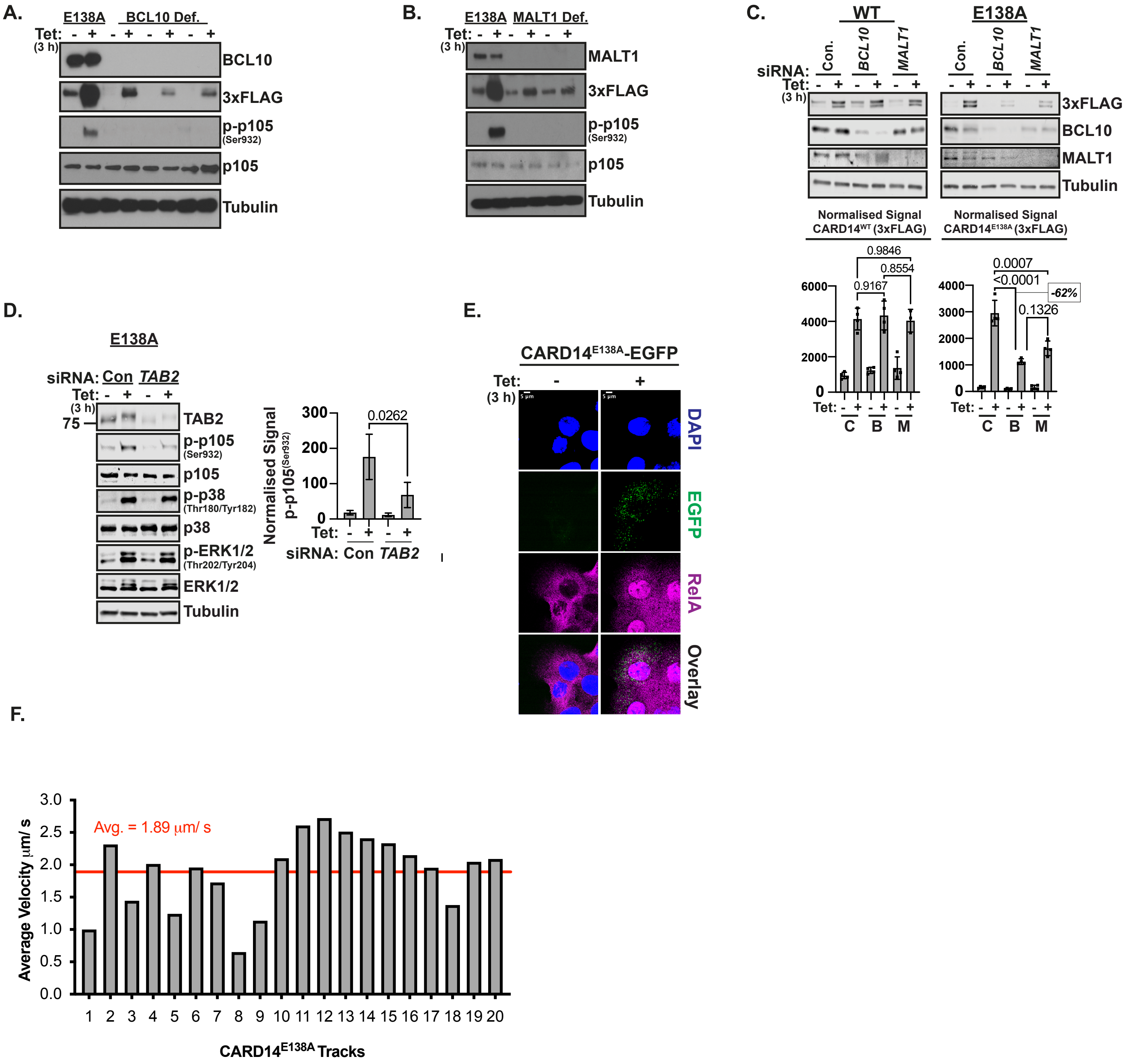

A.
E138A
BCL10 Def.
Tet:
(3 h)
BCL10
3xFLAG
p-p105 (Ser932)
p105
Tubulin
B.
E138A
MALT1 Def.
MALT1
p-p105 (Ser932)
C.
WT
E138A
siRNA:
Con.
BCL10
MALT1
Normalised Signal CARD14WT (3xFLAG)
Normalised Signal CARD14E138A (3xFLAG)
0.9846
0.8554
0.9167
0.0007
<0.0001
-62%
0.1326
C
B
M
D.
siRNA: Con TAB2
TAB2
p-p38 (Thr180/Tyr182)
p38
p-ERK1/2 (Thr202/Tyr204)
ERK1/2
Normalised Signal p-p105(Ser932)
0.0262
E.
CARD14E138A-EGFP
DAPI
EGFP
RelA
Overlay
F.
Avg. = 1.89 µm/ s
Average Velocity µm/ s
CARD14E138A Tracks

S5

A.

Table. Noteworthy Phosphosites

Gene	Position	AA	PMID	Mix 2	Mix 3
ATF7	51	T	21858082	-1.38	3.39
ATF7	53	T	21858082	-1.38	3.39
FOSL1	265	S	17371847	-2.55	3.33
FOSL2	320	S	25547114	-1.99	2.24
FOSL2	200	S	23807221	-0.57	0.77
JUN	63	S	14617628	-2.94	4.60
JUN	243	S	17482134	-2.01	1.34
JUN;JUND	73	S	1749429	-2.81	3.82
JUNB	251	S	22710716	-1.16	1.63
JUNB	259	S	22710716	-1.33	1.34
JUNB	255	T	22710716	-1.32	1.33
MAPK1	185	T	1378617	-1.79	2.03
MAPK1	187	Y	22569528	-1.80	2.03
RICTOR	1135	T	19995915	-0.82	1.82
RPTOR	863	S	19864431	-0.92	1.11
TAB2	372	S	18021073	-0.93	1.05
TNFAIP3	381	S	26649818	-2.93	1.88
TRAF2	11	S	18981220	-2.35	2.26

B.

p38 Inhibition

E138A

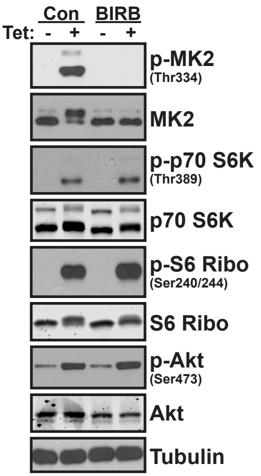

C.

MEK Inhibition

E138A

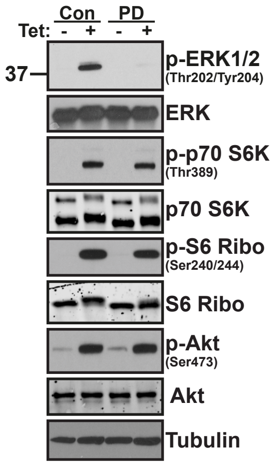

D.

JNK Inhibition

E138A

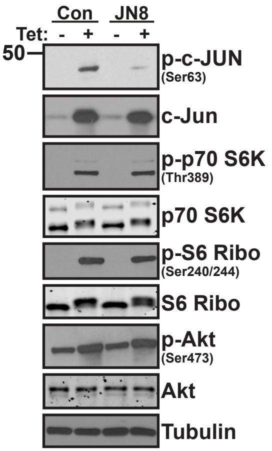

E.

MALT1 Inhibition

E138A

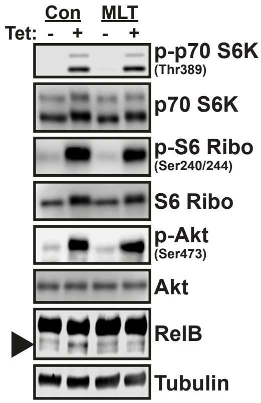

F.

mTORC1 Inhibition

E138A

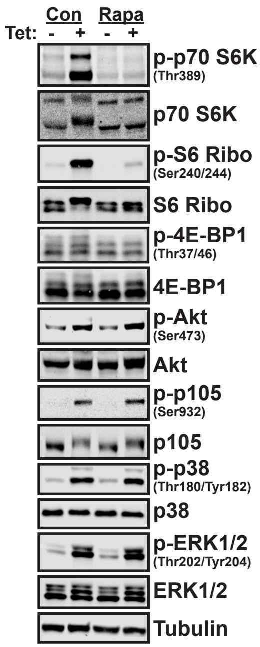

G.

Akt Inhibition

E138A

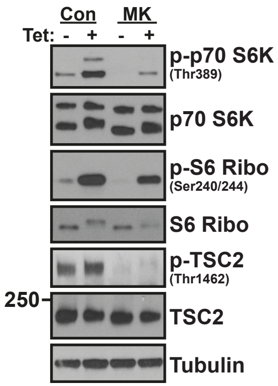

S6.

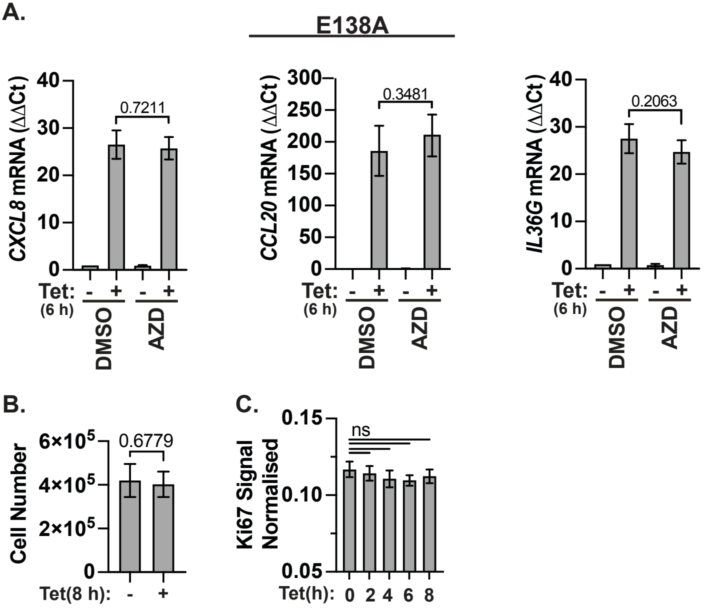
A.
E138A
CXCL8 mRNA (ΔΔCt)
0.7211
CCL20 mRNA (ΔΔCt)
0.3481
IL36G mRNA (ΔΔCt)
0.2063
Tet: - + - +
(6 h)
DMSO
AZD
B.
Cell Number
0.6779
Tet(8 h): - +
C.
Ki67 Signal Normalised
ns
Tet(h): 0 2 4 6 8

S7.

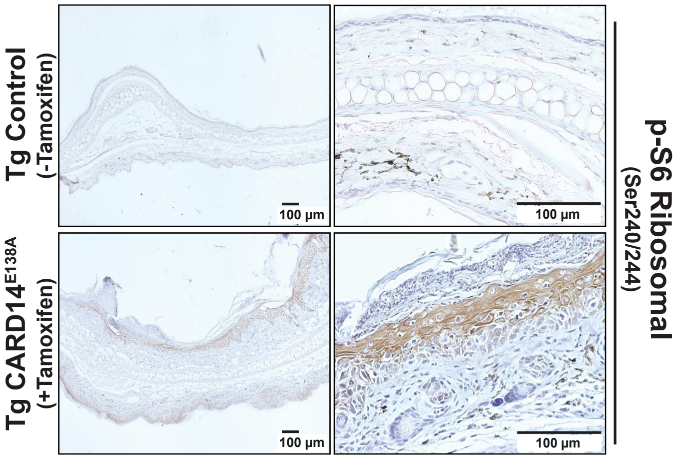

Tg Control
(-Tamoxifen)
Tg CARD14E138A
(+Tamoxifen)
p-S6 Ribosomal
(Ser240/244)
100 µm
100 µm
100 µm
100 µm
